# Hu14.18K.322A Causes Direct Cell Cytotoxicity and Synergizes with Induction Chemotherapy in High-Risk Neuroblastoma

**DOI:** 10.3390/cancers16112064

**Published:** 2024-05-30

**Authors:** Maria Thomas, Thu Hien Nguyen, Jenny Drnevich, Amber M. D’Souza, Pedro A. de Alarcon, Manu Gnanamony

**Affiliations:** 1 Department of Pediatrics, University of Illinois College of Medicine Peoria, One Illini Drive, Peoria, IL 61605, USA; tmaria@uic.edu (M.T.); thuhien@uic.edu (T.H.N.); dsom6v@uic.edu (A.M.D.); pdealarc@uic.edu (P.A.d.A.); 2 Roy J. Carver Biotechnology Center, The University of Illinois at Urbana-Champaign, 1206 W. Gregory Drive, Urbana, IL 61801, USA; drnevich@illinois.edu

**Keywords:** neuroblastoma, direct cell death, synergy

## Abstract

**Simple Summary:**

The disialoganglioside GD2 is a molecule found in certain tumors, like neuroblastoma, but rarely in normal tissues. The main aim of this study was to understand if hu14.18K322A, an antibody targeting GD2, causes direct cell death in high-risk neuroblastoma and to delineate the pathways involved. Our results demonstrate that hu14.18K322A activates apoptosis and necroptosis but affects no other cell-death pathways. Hu14.18K322A synergistically increased cell death by induction chemotherapy drugs. We found an alteration in GD2 surface expression after chemotherapy treatment, emphasizing the complex interplay between them. These results highlight the multifaceted role of hu14.18K322A in high-risk neuroblastoma and suggest pathways that may be targeted to increase treatment efficacy.

**Abstract:**

The disialoganglioside, GD2, is a promising therapeutic target due to its overexpression in certain tumors, particularly neuroblastoma (NB), with limited expression in normal tissues. Despite progress, the intricate mechanisms of action and the full spectrum of the direct cellular responses to anti-GD2 antibodies remain incompletely understood. In this study, we examined the direct cytotoxic effects of the humanized anti-GD2 antibody hu14.18K322A (hu14) on NB cell lines, by exploring the associated cell-death pathways. Additionally, we assessed the synergy between hu14 and conventional induction chemotherapy drugs. Our results revealed that hu14 treatment induced direct cytotoxic effects in CHLA15 and SK-N-BE1 cell lines, with a pronounced impact on proliferation and colony formation. Apoptosis emerged as the predominant cell-death pathway triggered by hu14. Furthermore, we saw a reduction in GD2 surface expression in response to hu14 treatment. Hu14 demonstrated synergy with induction chemotherapy drugs with alterations in GD2 expression. Our comprehensive investigation provides valuable insights into the multifaceted effects of hu14 on NB cells, shedding light on its direct cytotoxicity, cell-death pathways, and interactions with induction chemotherapy drugs. This study contributes to the evolving understanding of anti-GD2 antibody therapy and its potential synergies with conventional treatments in the context of NB.

## 1. Introduction

GD2 is a disialoganglioside overexpressed in NB, with restricted expression in normal tissues, making it an attractive therapeutic target [1]. GD2 has a pro-tumoral role in several cancers. In small cell lung cancer (SCLC) cell lines, GD2 induced growth and enhanced invasion abilities [2]. The last three decades have seen the introduction of several anti-GD2 antibodies for the treatment of high-risk NB [3,4]. The mechanism of action of this drug is through antibody-dependent cell cytotoxicity (ADCC) facilitated by NK cells, tumor-associated macrophages (TAMs) and complement-dependent cytotoxicity (CDC) [3,5,6]. There is also in vitro evidence that anti-GD2 antibodies have direct cytotoxic effects on NB cell lines [7]. The addition of anti-GD2 antibodies to the cell culture media resulted in the attenuation of growth and eventual cell death through the MAPK signaling pathway [8]. Mouse IgG antibody 3F8 induced direct cytotoxicity in GD2-presenting NB and melanoma cell lines but not in GD2-non-expressing colon cancer cells [9]. The humanized anti-GD2 antibody hu14.18K322A (hu14) was designed with a point mutation in hu14 to reduce complement activation [10]. A phase II trial that incorporated hu14 with induction-phase chemotherapy for newly diagnosed NB patients showed a better response rate than chemotherapy alone and will be studied in an upcoming phase III trial [11].

For a long time, apoptosis was considered the major programmed cell-death pathway in cells exposed to cytotoxic agents. Recently the existence of other cell-death pathways like ferroptosis, necroptosis, pyroptosis, parthanatos, and PANoptosis have gained prominence in cancer research [12,13,14,15,16]. Anti-GD2 mouse monoclonal antibody 14G2a was shown to induce direct cell cytotoxicity in certain NB cell lines [17], but the analysis was restricted to apoptosis as this was the major cell-death pathway known at that time.

In this study, we evaluated hu14-induced direct cell death in various NB cell lines and explored the various cell-death pathways activated in response to hu14. We studied the transcriptomic changes caused by the exposure of sensitive NB cells to hu14. Finally, we evaluated the synergy between hu14 and induction chemotherapy drugs and the role chemo drugs played on GD2 biosynthesis pathways.

## 2. Materials and Methods

### 2.1. Cell Lines and Media

We obtained CHLA15, SK-N-BE1, SK-N-BE2, LAN5 and LAN6 from the childhood cancer repository of the Children’s Oncology Group (COG). All cell lines were cultured in RPMI1640 media supplemented with 10% FBS and 1% penicillin streptomycin. Hu14.18K322A was provided by St. Jude Children’s research hospital. Human IgG (I4506) was purchased from Sigma and used as a control for all hu14 experiments. Mycoplasma testing was carried out on all growing cells periodically using Mycoplasma PCR Detection and Elimination Kits (ABM, Richmond, BC, Canada).

### 2.2. Cell Viability

We used the dye resazurin (Resazurin Cell Viability Assay Kit, Biotium, Fremont, CA, USA) to measure cell viability for all the experiments. Briefly, CHLA15 (7500) and SK-N-BE1 (10,000) cells were plated in 96-well plates for 24 h. After appropriate drug treatment, the plates were incubated in a CO_2_ incubator for 72 h. At the time of the assay, 10 μL of resazurin dye was added to the wells, incubated for an additional 4 h in the CO_2_ incubator. Plates were read at 450 nm with correction at 600 nm in an xMark Microplate Spectrophotometer (Bio-Rad, Hercules, CA, USA). Percent viability was calculated using Microsoft^®^ Excel^®^ for Microsoft 365 MSO (Version 2404 Build 16.0.17531.20152) 64-bit.

### 2.3. Proliferation Assay

CHLA15 (7500 per well) and SK-N-BE1 (30,000 per well) cells were plated in 96-well plates 24 h before treatment. Cells were treated with IgG (20 μg/mL) or hu14 (1–20 μg/mL) and incubated in 5% CO_2_ incubator at 37 °C. Cell growth was measured every day using resazurin reagent as described in the earlier section. Optical density (OD) was normalized to cell density on day 0.

### 2.4. Colony Formation Assay

Counted cells were treated with IgG (20 μg/mL) and hu14 (20 μg/mL), seeded at 2500 cells per well in 6-well plates and grown in 5% at 37 °C. Media was changed every 3 days until colonies were visible. The cells were washed with PBS and fixed using acetic acid–methanol at a ratio of 1:7 (*v*/*v*). The cells were then stained with 0.5% crystal violet for 10 min. The number of colonies formed were visually counted.

### 2.5. Cell Cycle Analysis

CHLA15 and SK-N-BE1 cells were plated for 24 h and then treated with drugs. After 24 h drug treatment, cells were harvested by trypsinization and were incubated with Hoechst 33342 stain at a concentration of 10 μg/mL in growth media for 90 min at 37 °C with intermittent mixing. The cell cycle phase distribution was then determined by flow cytometer (CytoFLEX, Beckman Coulter, Indianapolis, IN, USA) and the data were analyzed using Kaluza software (Beckman Coulter, version 2.2.00001.20183).

### 2.6. GD2 Surface Expression Quantitation

Cells were grown on 100 mm plates for 24 h pre-treatment. Cells were exposed to drug treatment for another 24 h. Cells were then harvested by trypsinization, washed, counted and 1 × 10^6^ cells were incubated with Alexa Fluor^®^ 647 mouse anti-human ganglioside GD2 antibody (Biolegend, San Diego, CA, USA) in 100 μL of cell staining buffer (Biolegend) at a concentration of 5 ng/μL. Alexa Fluor^®^ 647 Mouse IgG2a, κ (Biolegend) was used for isotype control. After incubating for 20 min on ice in the dark, the cells were washed twice and re-suspended in 500 μL of cell staining buffer. The cells were then incubated with 5 μL of DAPI solution for 2–3 min and fluorescence measured by CytoFLEX flow cytometer. The experiments were performed once, and three readings were taken. The data were analyzed using Kaluza software.

### 2.7. Western Blot Analysis

Total cellular protein was extracted using M-PER™ Mammalian Protein Extraction Reagent (Thermo Scientific, Waltham, MA, USA) supplemented with Halt™ protease and phosphatase inhibitors (Thermo Scientific). The extracts (30–40 μg per well) were resolved in 10% or 12% SDS-PAGE gels and were transferred to nitrocellulose membranes (Bio-Rad). The membranes were blocked using 5% nonfat dry milk prior to overnight incubation with primary antibodies at 4 °C. After washing, the membranes were incubated with corresponding secondary antibodies and developed using Pierce™ ECL Western Blotting Substrate (Thermo Scientific). Densitometric analysis of the blots was performed using Image J 1.53e. The list of antibodies used in this study are given in Supplementary File S1.

### 2.8. RNA Isolation and Real-Time PCR

Total RNA was isolated using Trizol reagent (Invitrogen, Waltham, MA, USA) and the concentration was estimated using Nanodrop 2000 (Thermo Scientific). cDNA was synthesized using High-Capacity cDNA Reverse Transcription Kit (Applied Biosystems, Waltham, MA, USA) and amplified by real-time PCR using the PowerUp SYBR Green Master Mix (Applied Biosystems) using the cycling conditions recommended by the kit manufacturer. Sequences of primers used for this study are given in Supplementary Table S1. Gene expression data were normalized against two housekeeping genes SDHA and HPRT [18].

### 2.9. Apotracker Assay for Apoptosis

Cells were harvested by trypsin treatment and washed twice in PBS. Cells were counted and 1 × 10^6^ cells from each sample were then incubated in the dark at room temperature with Apotracker™ Green (BioLegend) at a concentration of 200 nM and 1/500 dilution of Zombie Violet (BioLegend) made in a total volume of 100 μL of cell staining buffer (BioLegend). After 15 min of incubation, the cells were washed, re-suspended in 500 μL of cell staining buffer and analyzed in a CytoFLEX flow cytometer. Dot plots with quadrant gates were generated with fluorescence of Apotracker green on the X-axis and Zombie Violet on the Y-axis. The lower left quadrant shows healthy cell populations, the lower right quadrant shows early apoptotic cells, and the upper left and upper right quadrants show the necrotic/late apoptotic cells.

### 2.10. Lipid Peroxidation Analysis

SK-N-BE1 and CHLA15 (3 × 10^6^) cells were seeded in 100mm plates and incubated for 24 h. After appropriate drug treatment, the plates were incubated in a CO_2_ incubator for another 24 h. After drug treatment, the cells were incubated with BODIPY™ 581/591 C11 reagent at 2 μM for 2 h in a CO_2_ incubator. The cells were then harvested by trypsinization, washed twice with Hanks balanced salt solution (HBSS). The experiments were performed once, and three readings were taken. The cells were re-suspended in HBSS, and fluorescence measured on a CytoFLEX flow cytometer (Indianapolis, IN, USA). The data were analyzed using Kaluza software.

### 2.11. RNA-Seq Data Analysis

Sample processing: Total RNA from IgG and hu14 (10 μg/mL) treated cells was extracted using Trizol (Invitrogen) and DNase treated using DNase (Qiagen). RNA sequencing was performed at the Roy J. Carver Biotechnology Center, University of Illinois at Urbana–Champaign. RNA was purified, libraries were constructed using the Kapa Hyper Stranded mRNA library kit (Roche, Basel, Switzerland) and sequenced 100 nt single end on one SP lane using the NovaSeq 6000 sequencing platform (Illumina, San Diego, CA, USA). Reads were demultiplexed per sample using bcl2fastq (v2.20; Illumina) and then pseudo-aligned to NCBI’s Annotation Release 110 transcriptome using Salmon (v1.5.2) with the entire genome GRCh38 as the decoy sequence [19].

Differential gene expression analysis: All analyses from summation of counts to over-representation testing were carried out using R version 4.2.1 (Supplementary File S3). Counts were summed to the gene-level using tximport’s (v1.24.0) “bias corrected counts without an offset” method [20]. The two cell lines differed in the amount of biological variation and so were analyzed separately. Genes without at least 0.5 count per million in at least 3 replicates were filtered out, leaving 15,255 genes for SK-N-BE1 and 15,700 genes for CHLA15. Differential gene expression (DE) analysis was performed using the edgeR-quasi method using the equivalent of a paired *t*-test to correct for replicate batch effects. False discovery rate (FDR) correction was performed on the *p*-values. The need for paired *t*-tests reduced the power to detect differences after peforming FDR correction for ~15,000 genes, so for SK-N-BE1, the FDR threshold was set at 0.2 and for CHLA15, the FDR threshold was set at 0.5 to include more true positives at the expense of a higher false positive rate. Over-representation testing was carried out separately for up- and downregulated genes to see if they had more Gene Ontology’s Biological Process, Cellular Component and Molecular Function terms or KEGG pathways than would be expected due to chance (Fisher’s one-sided Exact test).

### 2.12. Synergy and Drug Sensitization Assays

For determination of synergy, cells were plated in 96-well plates and co-treated with increasing doses of hu14 and chemotherapy drugs (cisplatin, cyclophosphamide, doxorubicin, etoposide, topotecan and vincristine) for 72 h. Viability was measured using resazurin reagent. Optical density values were imported and analyzed using Combenefit software (version 2.021) [21]. For drug sensitization testing, we pre-treated cells with hu14 for 4 h, then added the respective chemotherapy drugs and measured viability after 72 h.

### 2.13. Statistical Analysis

All statistical analysis was performed, and graphs were generated using Graphpad Prism v9. For all quantitation, experiments were performed in at least three replicates and all data are shown as mean ± standard deviation, unless otherwise specified. Student’s *t*-test was used when comparing two groups. Correlation analysis between GD2 expression and viability assay was carried out using Pearson correlation coefficient. Two-way ANOVA with Šidák post hoc correction was used to analyze viability assays and gene expression data between wild-type cell lines. For all other multigroup analysis, one-way ANOVA was used with Dunnett post hoc correction. *p* value < 0.05 was considered statistically significant.

### 2.14. Data Availability

The codes used for RNA sequencing data are available in the Supplementary Materials (Supplementary File S3). All other data will be made available upon request from the corresponding author.

## 3. Results

### 3.1. GD2 Expression Varies between NB Cell Lines, but Does Not Influence the Susceptibility of the Cells to Hu14

**Figure 1:**
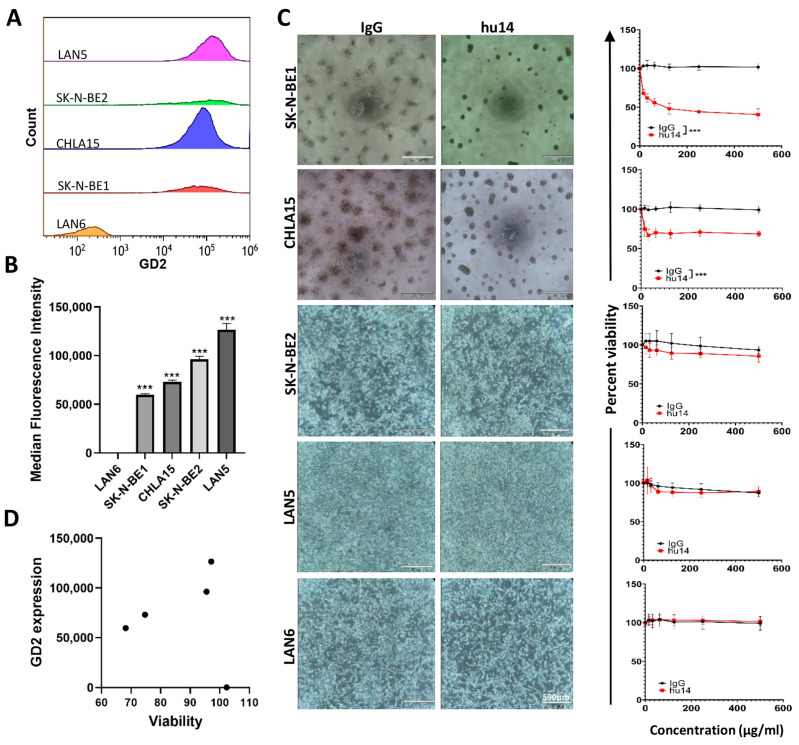
Hu14 treatment induces direct cell death in NB cells. (**A**) Representative flow cytometry image of GD2 surface expression in five NB cell lines. (**B**) Quantitation of the median fluorescence intensity of GD2 in NB cell lines. All cell lines showed significantly high GD2 expression compared to LAN6. ANOVA *** *p* < 0.001. (**C**) Representative 10X bright field images of IgG and hu14-treated NB cells showing aggregate formation, clumping and cell death in CHLA15 and SK-N-BE1 but no other cell lines in response to hu14 (Scale bar 590 μm) and corresponding graph showing survival after 72 h exposure to increasing concentrations (0−500 μg/mL IgG or hu14). Hu14-treated cells showed significant reduction in viability with all the concentrations tested in CHLA15 and SK-N-BE1 cell lines. ANOVA *** *p* < 0.001. (**D**) Graph showing lack of correlation between GD2 surface expression and cell viability r = −0.041, *p* = 0.948.

Using flow cytometry, we analyzed the surface expression of GD2 in five different cell lines (CHLA15, LAN5, LAN6, SK-N-BE1 and SK-N-BE2). Consistent with previous published studies, LAN6 cells were negative for GD2 expression whereas all the other four cell lines were positive (Figure 1A). The median fluorescence intensity of the GD2-positive cell population was highest for LAN5 cells followed by SK-N-BE2, CHLA15 and SK-N-BE1 (Figure 1B).

We then determined the susceptibility of the GD2 positive cell lines to hu14. Upon treatment with hu14, CHLA15 and SK-N-BE1 cells tend to aggregate and form clumps (Figure 1C). Based on visual observation, about 80% of these cells float by 72 h post treatment, whereas around 20% of the clumped cells remained attached to the bottom of the cell culture plate. This effect was not observed in LAN5 and SK-N-BE2 cells (Figure 1C). We did a resazurin viability assay to further understand the cytotoxic effect of hu14 on the NB cell lines. In CHLA15 and SK-N-BE1 cell lines, hu14 treatment significantly reduced cell viability in all concentrations tested whereas no change in viability was observed with isotype treatment *p* < 0.001. (Figure 1C). LAN5, LAN6 and SK-N-BE2 did not show any significant difference between the isotype treated and hu14-treated cells (Figure 1C). Surface expression levels of GD2 did not correlate with susceptibility to hu14 (Figure 1D). Since only CHLA15 and SK-N-BE1 were susceptible to hu14 treatment, subsequent experiments were performed only on these cell lines.

### 3.2. Hu14 Has an Anti-Proliferative Effect on Susceptible Cell Lines

**Figure 2:**
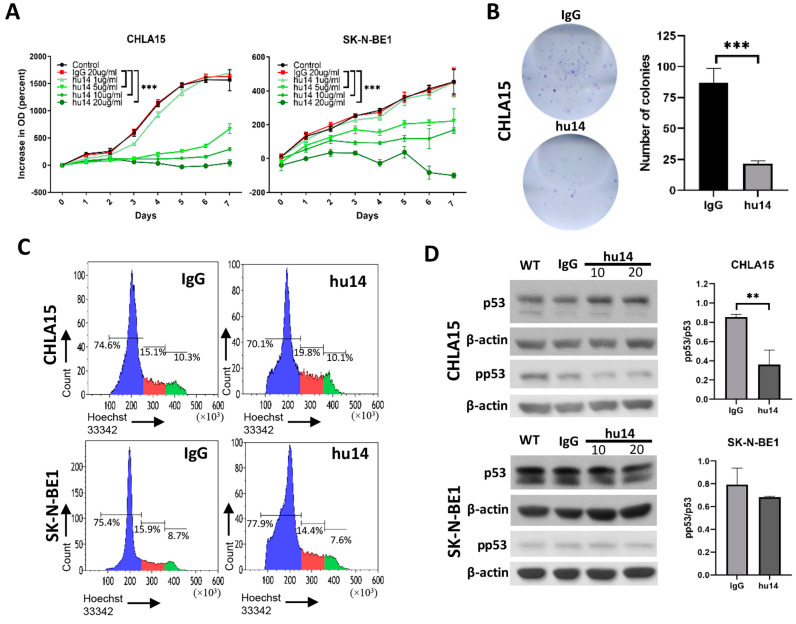
Hu14 blocks proliferation of sensitive NB cells. (**A**) Comparison of anti-proliferative effect of hu14 (1−20 μg/mL) with IgG (20 μg/mL) in CHLA15 and SK-N-BE1. ANOVA *** *p* < 0.001. (**B**) Representative image of colony formation in CHLA15 treated with hu14 (20 μg/mL) and IgG (20 μg/mL) and corresponding graph showing inhibition of colony formation by hu14. *t*-test *** *p* < 0.001. (**C**) Representative histogram plots showing cell cycle analysis of IgG (10 μg/mL) and hu14 (10 μg/mL) treated CHLA15 and SK-N-BE1 cells. (**D**) Representative Western blot image of p53 and phospho-p53 (pp53) from CHLA15 and SK-N-BE1 cells treated with hu14 (10 and 20 μg/mL). Graph showing pp53/p53 ratio normalized to β-actin between IgG (20 μg/mL) and hu14 (20 μg/mL) treatment analyzed by densitometry in CHLA15 and SK-N-BE1 cell lines. *t*-test (**D**). ** *p* < 0.01. The uncropped blots are shown in Supplementary File S2.

Next, we investigated the effect of different concentrations of hu14 antibody on the proliferation of CHLA15 and SK-N-BE1 cells. Hu14 showed a dose-dependent inhibitory effect on the proliferation of cells with 20 μg/mL causing the maximum inhibition in both the cell lines (Figure 2A). We then examined the effect of hu14 antibody (20 μg/mL) on the colony formation potential of the CHLA15 cells. The number of countable colonies was found to be significantly lower (*p* < 0.001) in hu14-treated cells compared to cells treated with isotype control antibody (Figure 2B).

To further understand the mechanism behind the anti-proliferative effect of the hu14 antibody, we performed cell cycle analysis by staining with Hoechst 33342. We found that hu14 treatment did not cause cell cycle arrest at any phases of the cycle in either CHLA15 or SK-N-BE1 cells (Figure 2C). This was supported by our Western blot data, which showed no change in protein levels of CDKN1B, cyclin D1, Rb and phospho-Rb in both CHLA15 and SK-N-BE1 cell lines after hu14 treatment (Supplementary Figure S1A,B). Upon hu14 treatment, we found a significant reduction in p53 phosphorylation ratio in CHLA15 but not SK-N-BE1 at 20 μg/mL concentration in comparison to isotype control (*p* < 0.01) (Figure 2D).

### 3.3. Apoptosis Is the Major Pathway Triggered by Hu14

**Figure 3:**
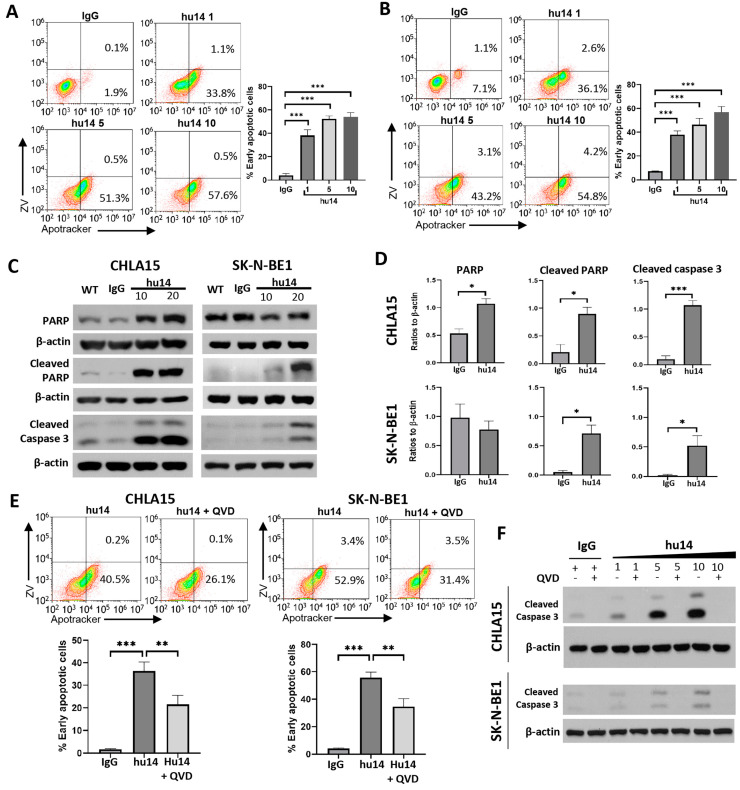
Hu14-induced apoptotic cell death in NB cells. CHLA15 (**A**) and SK-N-BE1 (**B**) cells were treated with hu14 (1, 5, 10 μg/mL) for 24 h and apoptosis/necrosis measured using Apotracker–Zombie Violet staining. Early apoptotic cells increased in a dose-dependent manner in hu14-treated cells. ANOVA *** *p* < 0.001. (**C**) Representative Western blot images of PARP, cleaved PARP, cleaved caspase 3 in response to hu14 (10, 20 μg/mL) in both CHLA15 and SK-N-BE1 cell lines. (**D**) Densitometric quantitation of the Western blot results show significant increase in cleaved PARP and cleaved caspase 3 in both CHLA15 and SK-N-BE1 in response to hu14 (20 μg/mL). *t*-test * *p* < 0.05, *** *p* < 0.001. (**E**) Representative graph showing flow cytometric analysis of CHLA15 and SK-N-BE1 cells treated with pan-caspase inhibitor QVD-OPH (10 μM) and hu14 and corresponding graph showing significant reversal of hu14-induced apoptotic cell death by QVD-OPH. ANOVA ** *p* < 0.01, *** *p* < 0.001. (**F**) Western blot images of caspase 3 showing dose-dependent increase in caspase 3 cleavage in both CHLA15 and SK-N-BE1 cell line. This effect is completely blocked by the addition of pan-caspase inhibitor QVD-OPH. The uncropped blots are shown in Supplementary File S2.

Recent understanding of cell death identified multiple pathways that can be potentially altered in response to chemotoxic agents, the most common being apoptosis. We measured apoptosis/necrosis using the Apotracker-FITC/Zombie Violet assay after 24 h treatment with hu14. Hu14 induced a dose-dependent increase in early apoptotic but not necrotic cells in both CHLA15 and SK-N-BE1 cell lines (Figure 3A,B). Western blot analysis showed a dose-dependent increase in the levels of cleaved PARP and cleaved caspase 3 in both cell lines (Figure 3C,D). PARP levels increased in CHLA15 but not SK-N-BE1 cell line in response to hu14. Hu14-induced apoptosis was reversed by QVD-OPH, a pan-caspase inhibitor as shown by Apotracker Green flow cytometry assay (Figure 3E) and Western blotting (Figure 3F). These results strongly prove that apoptosis is a major pathway of cell death in response to hu14.

### 3.4. Role of Other Cell-Death Pathways in NB Cells Treated with Hu14

**Figure 4:**
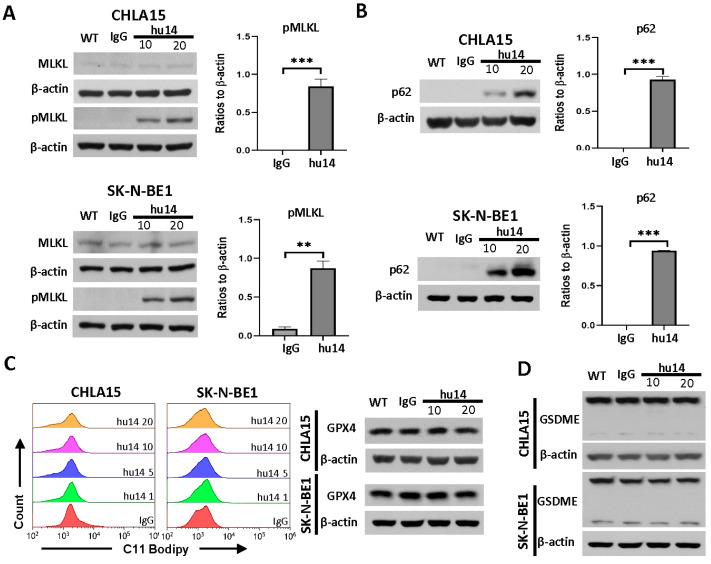
Role of necroptosis, autophagy, ferroptosis and pyroptosis in hu14-induced cell death. (**A**) Western blot image of CHLA15 and SK-N-BE1 cells showing MLKL and pMLKL in response to hu14 (10, 20 μg/mL) and corresponding graph showing significant increase in pMLKL in response to hu14 (20 μg/mL). *t*-test ** *p* < 0.01, *** *p* < 0.001. (**B**) Western blot image of p62 in response to hu14 (10, 20 μg/mL). Graph with densitometric analysis showing dose-dependent increase in p62 in response to hu14 (20 μg/mL). *t*-test *** *p* < 0.001. (**C**) Graph showing lipid peroxidation (BODIPY™ 581/591 C11 staining) in CHLA15 and SK-N-BE1 cells treated with various concentrations of hu14 (1–20 μg/mL). Hu14 did not induce lipid peroxidation in either cell line. GPX4 protein levels as shown by Western blot did not change upon hu14 (10, 20 μg/mL) treatment. (**D**) Western blot analysis of GSDME in CHLA15 and SK-N-BE1 cell line showing no change between IgG and hu14 (10, 20 μg/mL) treatment. The uncropped blots are shown in Supplementary File S2.

MLKL and RIPK3 are vital for the induction of necroptosis and are known to be suppressed or altered in many cancers [22]. In the NB cell lines that we tested, RIPK3 expression was absent and MLKL was faint except in LAN6 (Supplementary Figure S2A). In response to hu14, we found significant upregulation of phospho-MLKL (Figure 4A) but no change in MLKL or RIPK1 protein levels (Figure 4A and Figure S2B). In both CHLA15 and SK-N-BE1 cells, we did not find changes in the mRNA levels of MLKL and RIPK1 genes (Supplementary Figure S2C). pRIPK1 and pRIPK3 levels were not detected in response to hu14. These results suggest that either MLKL-mediated necroptosis is activated by an unknown mechanism or phospho-MLKL has functions other than inducing necroptotic cell death.

Autophagy flux can be studied by measuring LC3-II increase in the presence of bafilomycin A1 and p62 decrease in the absence of bafilomycin A1 using Western blot [23]. We found no induction of LC3-II expression but a dose-dependent increase in p62 (*p* < 0.001) (Figure 4B and Figure S2D,E). These results suggest that autophagy may be inhibited by hu14 treatment.

We measured changes in lipid peroxidation using BODIPY™ 581/591 C11 staining reagent to study ferroptosis. Hu14 did not induce lipid peroxidation in either the CHLA15 or SK-N-BE1 cell line to increasing concentrations of hu14 (Figure 4C). The protein level of GPX4, a marker of ferroptosis, was not altered in response to hu14 (Figure 4C). In addition, mRNA levels of PTGS2 (a known marker of ferroptosis) and GPX4 did not change in response to hu14 (Supplementary Figure S2C). These results suggest that ferroptosis is not a major pathway in hu14-induced cell death.

We measured the mRNA levels of the gasdermin family genes (GSDMA, GSDMB, GSDMC, GSDMD and GSDME) in all five wild-type NB cell lines to understand the genes activated in neuroblastoma. Among the cell lines tested, GSDME was the highest expressed member of the family (*p* < 0.001) (Supplementary Figure S2F). Therefore, we measured the changes in GSDME activation in CHLA15 and SK-N-BE1 after hu14 treatment. We did not observe any change in levels of N-GSDME, a marker of pyroptosis using Western blot, suggesting that this pathway is not activated in response to hu14 (Figure 4D).

### 3.5. Effect of Hu14 on GD2 Biosynthesis

**Figure 5:**
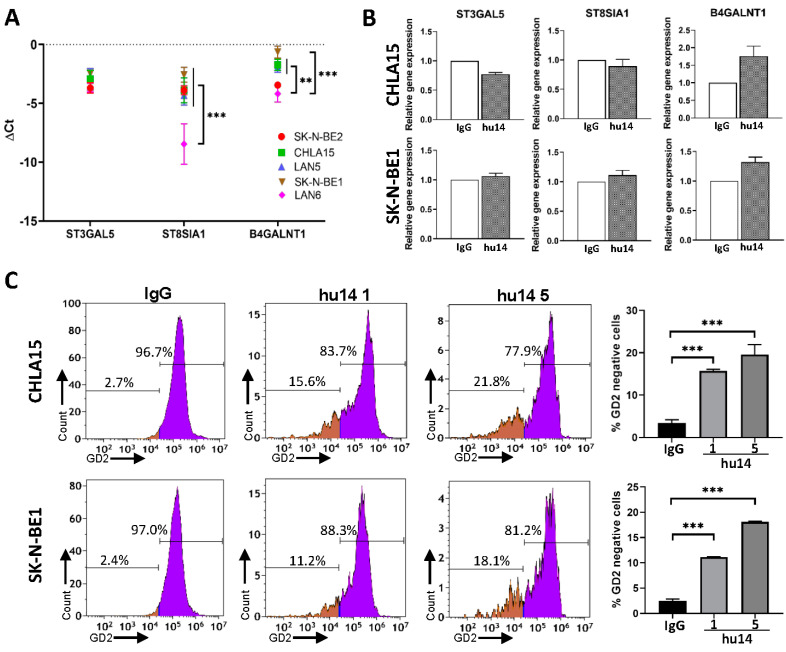
Effect of hu14 on GD2 synthesis and expression. (**A**) Real-time PCR analysis of GD2 pathway gene expression shown as mean ± SD in five wild-type NB cell lines. ANOVA ** *p* < 0.01, *** *p* < 0.001. (**B**) Comparison of relative expression of GD2 pathway genes in hu14 (10 μg/mL)-treated cells and IgG (10 μg/mL)-treated CHLA15 and SK-N-BE1 cell lines. (**C**) Representative histograms showing GD2 expression in cells treated with hu14 (1 μg and 5 μg/mL) for 24 h. GD2-negative populations are shown in brown. Corresponding graph showing a dose-dependent increase in GD2-negative cells in response to hu14. ANOVA *** *p* < 0.001.

To understand the role of the GD2 biosynthesis pathway on GD2 expression, we first measured the expression of the genes involved in the GD2 biosynthesis pathway (ST8SIA1, ST3GAL5 and B4GALNT1) in all five NB cell lines using real-time PCR. The GD2 negative cell line LAN6 had significantly lower expression of ST8SIA1 compared to other GD2-positive cell lines (*p* < 0.001) (Figure 5A). B4GALNT1 levels were significantly lower in LAN6 than in other cell lines except SK-N-BE2 (*p* < 0.05, *p* < 0.001) (Figure 5A). Hu14 treatment did not alter mRNA expression of the GD2 pathway genes in either CHLA15 or SK-N-BE1, suggesting alternate mechanisms of control (Figure 5B). However, hu14 significantly reduces GD2 surface expression in both CHLA15 and BE1 cell lines as seen by flow cytometry. (*p* < 0.001) (Figure 5C).

### 3.6. Transcriptional Changes Induced by Hu14 in CHLA15 and SK-N-BE1 Cells

**Figure 6:**
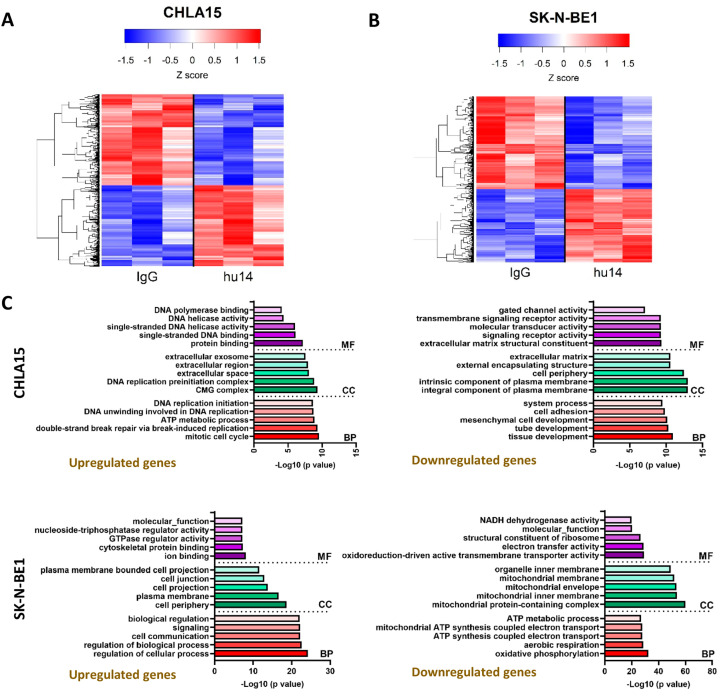
Hu14 alters transcriptome in NB. Heatmap showing gene expression changes in hu14-treated CHLA15 (**A**) and SK-N-BE1 (**B**) cells. Blue represents downregulated genes and red shows upregulated genes in fold change. GO enrichment analysis shows upregulated and downregulated genes in the top five categories in CHLA15 and SK-N-BE1 (**C**) cell lines. BP = biological process (shown in red), CC = cellular component (shown in green), MF = molecular function (shown in purple). RNA sequencing analysis was performed on three biological replicates for all treatment groups in both CHLA15 and SK-N-BE1 cell lines.

To identify transcriptional changes in the hu14-sensitive cell lines, we analyzed differentially expressed genes (DEGs) between hu14-treated and IgG-treated cells. In response to hu14 treatment, 635 genes were downregulated, and 586 genes were upregulated in CHLA15 cells (Figure 6A); 873 genes were downregulated, and 690 genes were upregulated in SK-N-BE1 (Figure 6B). We validated a select few genes in the RNA seq data using real-time PCR (Supplementary Figure S3A,B).

Gene ontology (GO) analysis in SK-N-BE1 indicated the downregulation of genes involved in mitochondrial structure, function and respiration, and the upregulation of cell communication, cell junction activity genes. In the CHLA15 cell line, the genes involved in DNA replication, protein binding and extracellular exosomes were upregulated and the genes involved in plasma membrane structure and signaling were commonly downregulated. The top five enriched GO terms, divided into biological processes (BPs), cell components (CCs) and molecular function (MF), are shown in Figure 6C.

KEGG pathway analysis showed different pathways altered in CHLA15 and SK-N-BE1 cells. While pathways linked to neurodegenerative diseases were significantly downregulated in SK-N-BE1 (*p* < 0.001), they were found to be upregulated in CHLA15 (Supplementary Table S2). Cancer growth and signaling pathways were upregulated in the SK-N-BE1 cell line but not in CHLA15 (Supplementary Table S2). The top 10 upregulated and downregulated genes in both the cell lines are listed in Supplementary Table S3.

### 3.7. Hu14 Synergizes with Induction Chemotherapy Drugs

**Figure 7:**
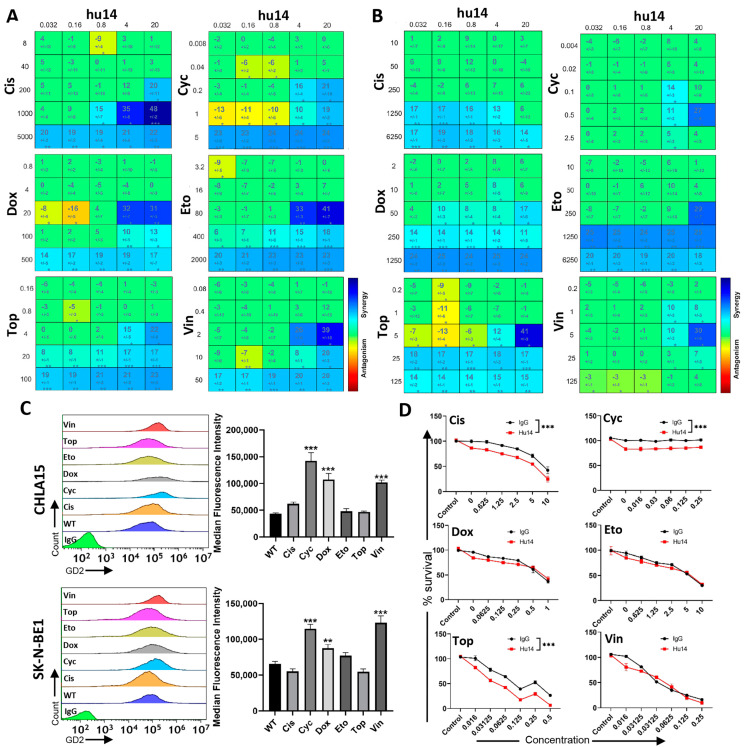
Hu14 synergizes with induction chemotherapy drugs. Plots from combenefit software showing synergy/antagonism between hu14 (0.032−20 μg/mL) in the x-axis and six drugs in the y-axis in CHLA15 (**A**) and SK-N-BE1 (**B**) cell lines. * *p* < 0.05, ** *p* < 0.01, *** *p* < 0.001. (**C**) Overlay histogram plots showing GD2 expression after drug treatment in CHLA15 and SK-N-BE1 cells. Graphs shows median fluorescent intensity of GD2 in response to six drugs. ANOVA ** *p* < 0.01, *** *p* < 0.001. (**D**) Survival graph showing SK-N-BE2 cells pre-treated with IgG/hu14 for 4 h and then treated with drugs for 72 h. Hu14 sensitized SK-N-BE2 cells to cisplatin, cyclophosphamide and topotecan but no other drugs. ANOVA *** *p* < 0.001. Cis = cisplatin (μM), Cyc = cyclophosphamide (μg/mL), Dox = doxorubicin (μM), Eto = etoposide (μM), Top = topotecan (μM), Vin = vincristine (μM).

Co-treatment of hu14 with six cycles of induction chemotherapy improved response rates and survival [11]. In our study, hu14 showed significant synergy with all six drugs (cisplatin, cyclophosphamide, doxorubicin, etoposide, topotecan and vincristine) in both CHLA15 and SK-N-BE1 cell lines (Figure 7A,B). To find out if chemotherapy drugs alter the surface expression of GD2, we treated CHLA15 cells with six drugs individually for 24 h and measured GD2 surface expression using flow cytometry. Cyclophosphamide, doxorubicin, and vincristine treatment significantly increased GD2-surface expression in both cell lines (*p* < 0.01) (Figure 7C). Next, we measured the effect of these six drugs on the expression of genes involved in the GD2 synthesis pathway—ST3GAL5, ST8SIA1, B4GALNT1. There was no change in the mRNA expression of these genes in CHLA15 in response to the six drugs (Supplementary Figure S4). We then tested whether pre-treatment with hu14 could enhance the cytotoxic effects of chemotherapy drugs in hu14-resistant cell lines. Pre-treatment (4 h) with hu14 significantly increased cell death induced by cisplatin, cyclophosphamide and topotecan with all the concentrations tested but not doxorubicin, etoposide, and vincristine in the SK-N-BE2 cell line, *p* < 0.001 (Figure 7D).

## 4. Discussion

We included a panel of cell lines that have a varied molecular profile to study the effects of hu14. CHLA15 and LAN6 are MYCN non-amplified and have a functional p53. SK-N-BE1 and LAN5 are MYCN amplified with a functional p53. SK-N-BE2 is MYCN amplified with a non-functional p53. Our results show that hu14-susceptible NB cell lines express GD2 expression at different levels; however, expression per se does not correlate with direct cell cytotoxicity. This suggests that GD2 expression is necessary but not enough for the direct effects of hu14 and that additional mechanisms must play a role. Tibbetts et al. demonstrated that ADCC induced by 14G2a, a mouse monoclonal antibody targeting GD2, had no correlation with GD2 expression [24]. They further showed that antibody internalization was inversely correlated with ADCC, suggesting a role for GD2-independent pathways responsible for the antibody-induced cytotoxic effect. Exposure to hu14 resulted in clumping and aggregate formation in our study. Similar effects were observed by Horwacik et al. in NB cells treated with 14Ga, suggesting that integrins and extracellular matrix might play a role in this phenomenon [25].

Much of the focus of immunotherapy is on optimizing ADCC and complement dependent cytotoxicity (CDC). The idea that antibodies themselves can cause direct cytotoxicity is interesting and may play a role in standardizing therapy. Direct cell cytotoxicity by anti-GD2 antibodies has been reported by other researchers over the years using 14G2a or 3F8, both anti-mouse monoclonal antibodies [9,25,26]. In this study, we investigated the mechanisms of direct cytotoxicity of a humanized anti-GD2 antibody, hu14.18K322A (hu14).

Hu14 did not alter the cell cycle pattern in either CHLA15 or SK-N-BE1 cell lines in our study. These data are supported by our Western blot analysis showing no changes in cell cycle protein levels (CDKN1B, Rb, p-Rb). This contrasts with Cochonneau et al. who showed a G1 and G2M phase arrest with a significant lack of S phase in response to 8B6 and 10B8 on O-acetylated GD2 ganglioside positive tumors [27]. The authors also showed an increase in p21, a marker of cell cycle arrest. We were not able to detect p21 in our study. p53 is a tumor-suppressor protein that plays an important role in cell cycle, proliferation, and cell death [28]. Horwacik et al. showed an increase in p53 levels in response to anti-GD2 antibody [25]. However, p53 decreased in SK-N-BE1 and remained unchanged in CHLA15 upon hu14 treatment in this study. Interestingly, we saw a decrease in p53 phosphorylation at serine 6 in response to hu14 in CHLA15 but not in the SK-N-BE1 cell line. Reduction in phosphorylated p53 levels maybe a tumor resistance mechanism to prevent further cell death and promote survival. Mirroring our results of high phosphorylated p53 in untreated controls, tumor derived cell lines have been shown to express high phosphorylation of p53 [29]. Both CHLA15 and SK-N-BE1 cell lines have a functional p53. p53 is phosphorylated at ser 6 in response to DNA-damaging drugs [30]. p53 can be phosphorylated at several positions and the extensive analysis of multiple phosphorylation sites will shed more light into its role in this aspect.

Among the pathways analyzed, apoptosis was the only pathway triggered in response to hu14. Interestingly, most of the dying cells were in the early stages of apoptosis, as shown in Figure 3. Even higher concentrations of hu14 did not induce necrosis at a 24 h time point. Longer exposure to the drug may lead to necrosis. Western blot analysis shows an increase in cleaved caspase 3 and cleaved PARP expression, both hallmarks of apoptosis. Thus, conforming to several other studies with anti-GD2 antibodies, we show that apoptosis is a major cell-death pathway associated with hu14 [7].

Necroptosis is a caspase-independent non-apoptotic cell death mechanism that involves RIPK1, RIPK3 and MLKL [31]. RIPK3 has been shown to be epigenetically silenced in many cancers. The oncogene MYC has been shown to block RIPK1 and RIPK3 interaction and thereby prevent necroptosis and the loss of MYC sensitizes cells to necroptosis [32]. Very few data are available on necroptosis in NB [33]. Our results show that MLKL is activated in both CHLA15 and SK-N-BE1. We also show that NB cells express RIPK1 and MLKL (faint) in all cell lines, but do not express even basal levels of RIPK3. RIPK3-mediated activation of MLKL is necessary for canonical necroptotic cell death [34]. However, MLKL has been shown to have necroptosis-independent roles. For example, MLKL increases vascular inflammation in endothelial cells by regulating ICAM1, VCAM1 [35]. Yoon et al. have shown that MLKL regulates endosomal trafficking independent of its role in necroptosis [36]. Activation of MLKL in the absence of RIPK3 in our study suggests that it might have alternate roles.

Ferroptosis is an iron-dependent non-apoptotic cell death mechanism [37]. Recent studies have shown that high-risk MYCN-amplified NB are susceptible to ferroptosis [38,39]. In this study, we did not find an increase in lipid peroxidation in response to hu14, suggesting that ferroptosis was not induced. These data were supported by our Western blot analysis of GPX4 and RNA markers of ferroptosis genes.

In our study, hu14 did not induce LC3B-II which is a marker of autophagic flux. This contrasts with Durbas et al. who showed that 14G2a mAb increased autophagic flux in a NB cell line [40]. However, we did see an increase in p62 levels which can be a marker of autophagy inhibition among other functions [41]. Blocking autophagy may be a survival mechanism used by NB cells in response to hu14. Further characterization of the autophagy dependent/independent roles of p62 is necessary.

Three of the most important genes that play a role in GD2 synthesis are ST3GAL5, B4GALNT1 (GD2 synthase) and ST8SIA1 (GD3 synthase) [42]. We found that the GD2-negative cell line LAN6 had a significantly lower expression of ST8SIA1 and B4GALNT1 which are key enzymes required for making GD2. The role of ST8SIA1 expression levels as a novel biomarker to identify GD2 therapy-resistant primary tumors needs to be explored further. Hu14 treatment was selected for GD2-negative cells in both cell lines, but no change in the expression of GD2-pathway genes was observed in this study. This suggests post-transcriptional mechanisms are involved in the regulation of GD2 expression on the cell surface.

Phenotypically, both CHLA15 and SK-N-BE1 cells showed a similar inhibition of cell growth and cell death pattern in response to hu14. However, when their transcriptome was analyzed by next-generation sequencing, we found that they activate completely opposite molecular pathways. The MYCN amplification difference between these cell lines may explain this difference.

Furman et al. showed that concomitant administration of hu14 with induction chemotherapy increased the survival outcome in high-risk NB, suggesting synergism between them [11]. Induction chemotherapy for high-risk NB has six cycles of a combination of cyclophosphamide, topotecan, cisplatin, doxorubicin, and vincristine [11]. Knowing if hu14 works well with all the six drugs is crucial in order to understand and optimize the therapy response. In this study, we studied synergy/additive/antagonism between hu14 and all six drugs. We found that in both hu14-sensitive cell lines, there was excellent synergy between hu14 and all six drugs. In addition, we showed that pre-treatment with hu14 sensitized a resistant cell line to treatment with some chemo drugs but not others. One way for increased synergistic cell death could be by the upregulation of GD2 on the cell surface. Kroesen et al. showed that vorinostat increased surface expression of GD2 as a possible mechanism of increased synergy with anti-GD2 mAb [43]. Troschke-Meurer showed that dinutuximab beta plus chemo drugs increased ADCC in a spheroid model [44]. In SCLC cell lines that express GD2, Yoshida et al. showed that an anti-GD2 mAb enhanced the cytotoxic effects of several anti-cancer drugs 2.4- to 7.8-fold [2].

None of the six drugs changed the expression of the GD2 pathway genes in CHLA15. Reduced ST8SIA1 expression decreased stemness in glioblastoma [45], whereas the high expression of ST8SIA1 is associated with enhanced tumorigenicity in breast cancer [46,47].

As a limitation, our analysis was performed mostly after 24 h exposure to the drug by which time the decision to trigger cell death had already been made. Perhaps analysis at different time points may identify other pathways.

## 5. Conclusions

The results from our study show that the humanized anti-GD2 antibody hu14.18K322A induces direct cell cytotoxicity in a subset of NB cells by activating apoptosis. Induction chemotherapy drugs synergize with hu14 and alter GD2 surface expression and can sensitize cells to chemotherapy. We demonstrate the varying susceptibility to hu14 treatment of different NB cell lines, indicating the necessity for personalized treatments tailored to individual patients. Translating these findings into 3D models or patient-derived xenografts holds promise for clinically relevant personalized therapy options in the future.

## Data Availability

The data presented in this study are available within the article and the Supplementary Materials.

## Supplementary Materials

The following supporting information can be downloaded at: https://www.mdpi.com/article/10.3390/cancers16112064/s1, Figure S1. Representative Western blot images of CDKN1B, cyclin D1, Rb and p-Rb in CHLA15 (A) and SK-N-BE1 (B) treated with hu14 (10, 20 μg/mL); Figure S2. (A) Representative Western blot image showing MLKL and RIPK1 protein levels in five wild-type NB cell lines. (B) Representative Western blot image showing RIPK1 protein expression in CHLA15 and SK-N-BE1 cells in response to hu14 (10, 20 μg/mL). (C) mRNA levels of PTGS2, GPX4, RIPK1 and MLKL in response to hu14 in CHLA15 and SK-N-BE1 cell lines. (D) Western blot image of LC3B in CHLA15 treated with hu14 (10 and 20 μg/mL) with or without bafilomycin A1 (2 nM). (E) Western blot image of LC3B in SK-N-BE1 treated with hu14 (10 and 20 μg/mL) with or without bafilomycin A1 (2 nM). (F) Relative gene expression of gasdermin family genes in all five NB cell lines tested. ANOVA *** *p* < 0.001; Figure S3. Real-time PCR validation of selected upregulated (A) and downregulated (B) genes from the RNA sequencing data in CHLA15 and SK-N-BE1 cell lines. * *p* <0.05, ** *p* < 0.01; Figure S4. Real-time PCR analysis of GD2 pathway genes in CHLA15 cell line treated with six drugs. Cis = cisplatin (μM), Cyc = cyclophosphamide (μg/mL), Dox = doxorubicin (μM), Eto = etoposide (μM), Top = topotecan (μM), Vin = vincristine (μM); Table S1. Primer sequences used in this study; Table S2. KEGG pathway enrichment analysis in response to hu14 treatment; Table S3. Genes altered in response to hu14; File S1: Supplemental methods; File S2: Raw WB images and densitometry intensity ratios.
