# Supplementary material for: Hu14.18K.322A Causes Direct Cell Cytotoxicity and Synergizes with Induction Chemotherapy in High-Risk Neuroblastoma
Source: Cancers (Basel). 2024 May 30;16(11):2064. doi: 10.3390/cancers16112064 (PMC11171330; doi:10.3390/cancers16112064)
Supplement: Supplementary file 1 [file cancers-16-02064-s001.zip › File S1-Supplemental methods.pdf]

Details of antibodies used in the study.

Name (normal)	Name (Full name)	Cat no.	Manufacturer
p53-mouse	p53 Antibody (DO-1)	sc-126	Santa Cruz Biotechnology
p-p53 rabbit	Phospho-p53 (Ser6) Antibody	9285	Cell Signaling Technology
PARP-rabbit	PARP (46D11) Rabbit mAb	9532	Cell Signaling Technology
Cleaved PARP-mouse	Cleaved-PARP (Asp214) (E2T4K) Mouse mAb	32563	Cell Signaling Technology
Cleaved caspase-rabbit	Cleaved Caspase-3 (Asp175) Antibody	9661	Cell Signaling Technology
Caspase 3- rabbit	caspase-3 Antibody (H-277)	sc-7148	Santa Cruz Biotechnology
MLKL-rabbit	MLKL (D2I6N) Rabbit mAb	14993	Cell Signaling Technology
pMLKL-rabbit	Phospho-MLKL (Ser358) (D6H3V) Rabbit mAb	91689	Cell Signaling Technology
P62-rabbit	SQSTM1/p62 (D5E2) Rabbit mAb	8025	Cell Signaling Technology
LC3B-mouse	LC3B (E5Q2K) Mouse mAb	83506	Cell Signaling Technology
GPX4-rabbit	GPX4 Antibody	52455	Cell Signaling Technology
Gasdermin E rabbit	Gasdermin E (E2X7E) Rabbit mAb	19453	Cell Signaling Technology
p27 mouse	CDKN1B/Kip1 p27 Antibody (F-8)	sc-1641	Santa Cruz Biotechnology
Cyclin D1 mouse	cyclin D1 Antibody (A-12)	sc-8396	Santa Cruz Biotechnology
Rb-mouse	Rb Antibody (IF8)	sc-102	Santa Cruz Biotechnology
pRb-rabbit	Phospho-Rb (Ser780) Antibody	9307	Cell Signaling Technology
RIP-rabbit	RIP (D94C12) XP® Rabbit mAb	3493	Cell Signaling Technology
b-Actin-mouse	beta Actin Antibody (C4)	sc-47778	Santa Cruz Biotechnology
b-Actin-rabbit	Beta Actin Polyclonal antibody	20536-1-AP	Proteintech
pRIP	Phospho-RIP (Ser166) (D1L3S) Rabbit mAb	65746	Cell Signaling Technology
RIP3	RIP3 (E1Z1D) Rabbit mAb	13526	Cell Signaling Technology
pRIP3	Phospho-RIP3 (Ser227) (D6W2T) Rabbit mAb	93654	Cell Signaling Technology

p21	Waf1/Cip1/CDKN1A p21 Antibody (F-5)	sc-6246	Santa Cruz Biotechnology

Sex of cell lines used in this study.

LAN5	Male
LAN6	Male
SK-N-BE1	Male
SK-N-BE2	Male
CHLA15	Female
