# Supplementary material for: Hu14.18K.322A Causes Direct Cell Cytotoxicity and Synergizes with Induction Chemotherapy in High-Risk Neuroblastoma
Source: Cancers (Basel). 2024 May 30;16(11):2064. doi: 10.3390/cancers16112064 (PMC11171330; doi:10.3390/cancers16112064)
Supplement: Supplementary file 1 [file cancers-16-02064-s001.zip › File S2-Raw WB images and densitometry intensity ratios.pdf]

Fig.2D

CHLA15 p53

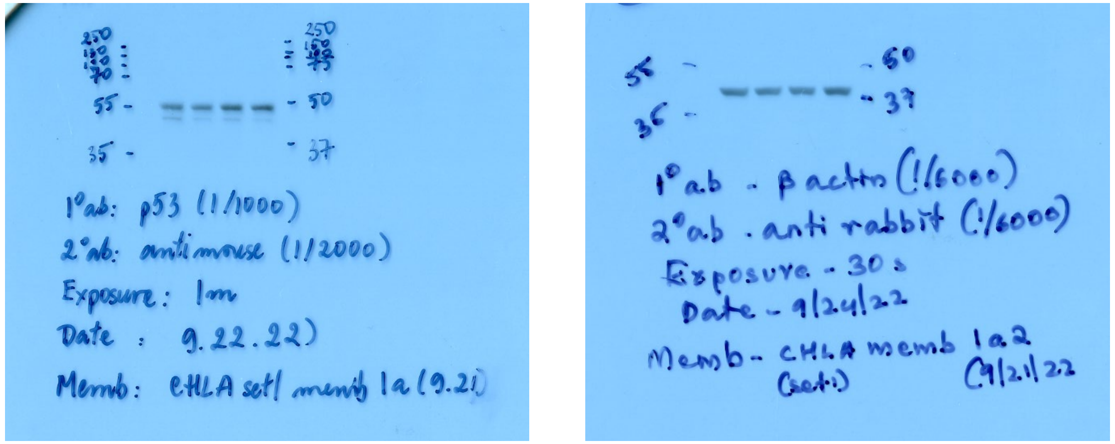

SK-N-BE1 p53

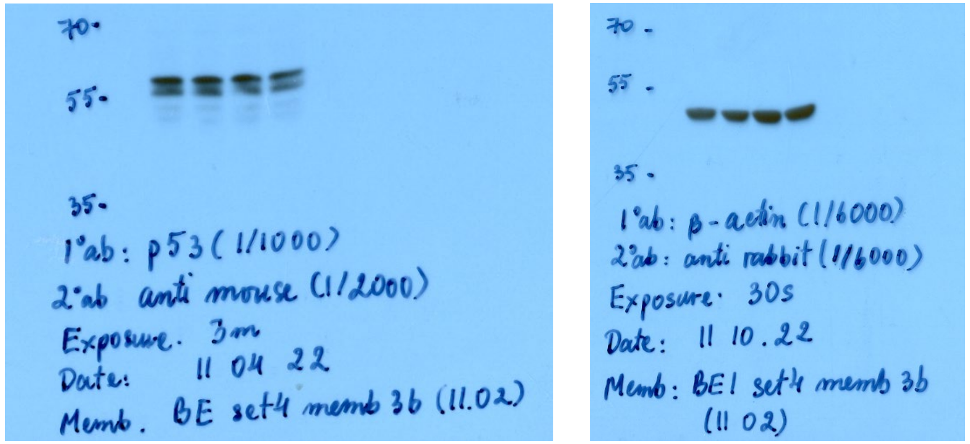

CHLA15 pp53

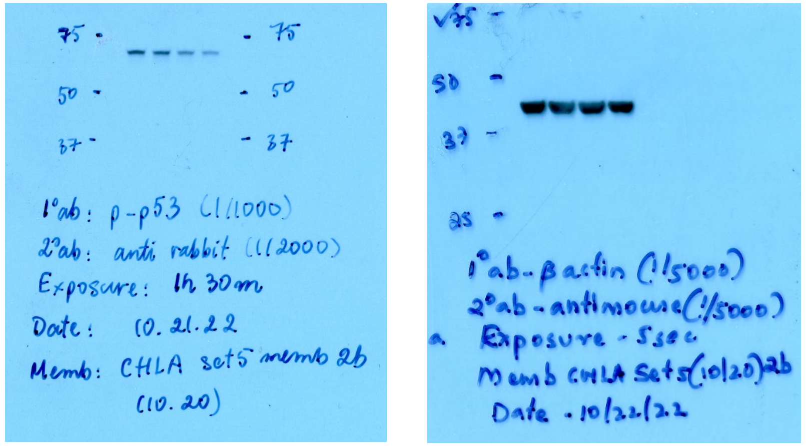

SK-N-BE1 pp53

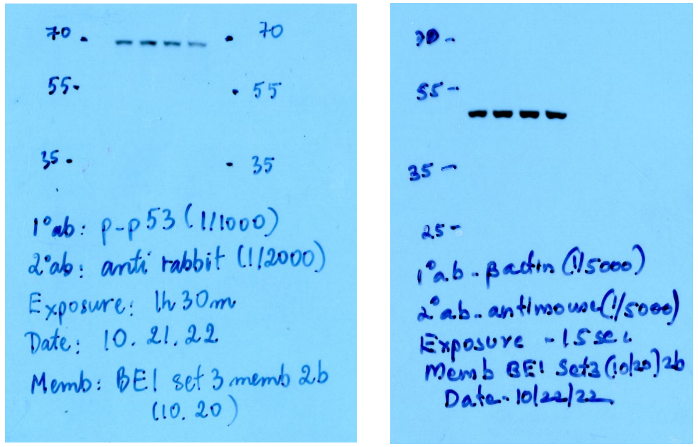

Fig.3C

CHLA15 PARP

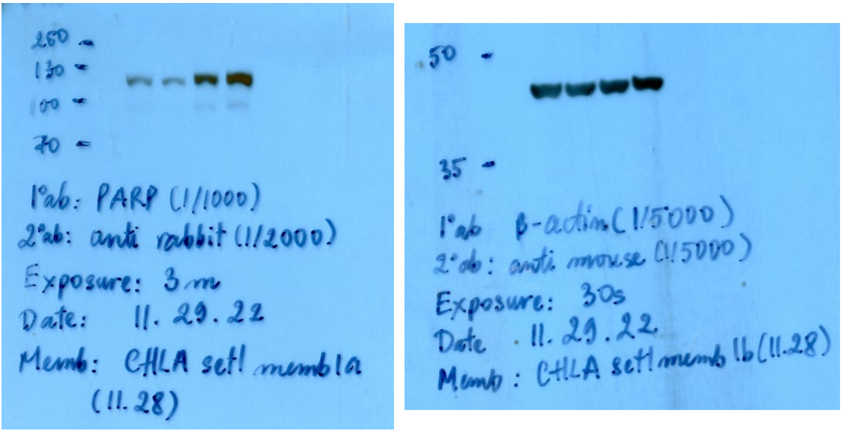

CHLA15 CLEAVED PARP

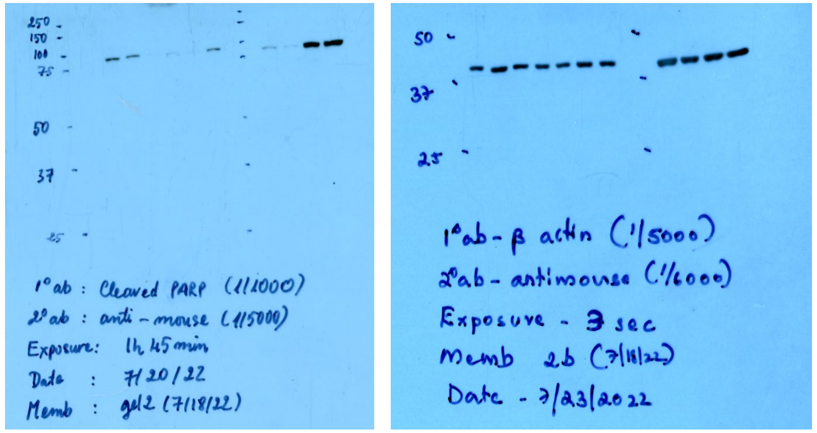

CHLA15 CLEAVED CASPASE 3

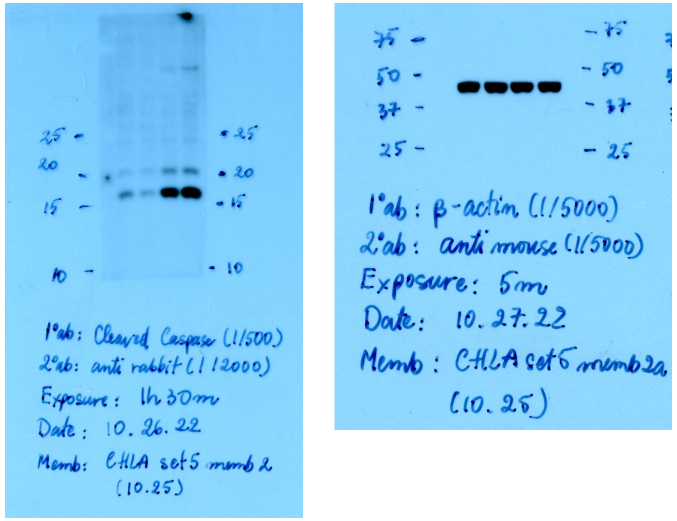

SK-N-BE1 PARP

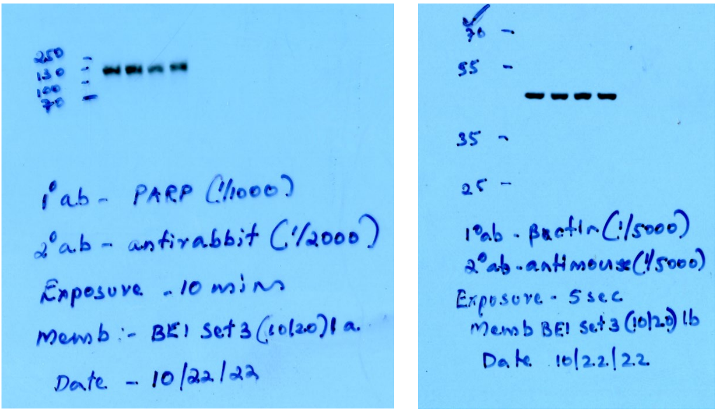

SK-N-BE1 CLEAVED PARP

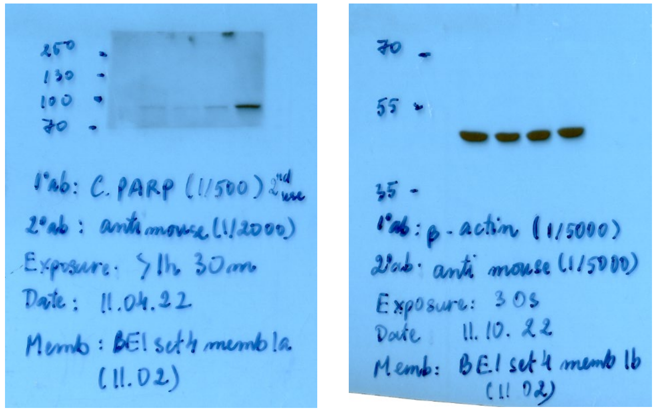

SK-N-BE1 CLEAVED CASPASE 3

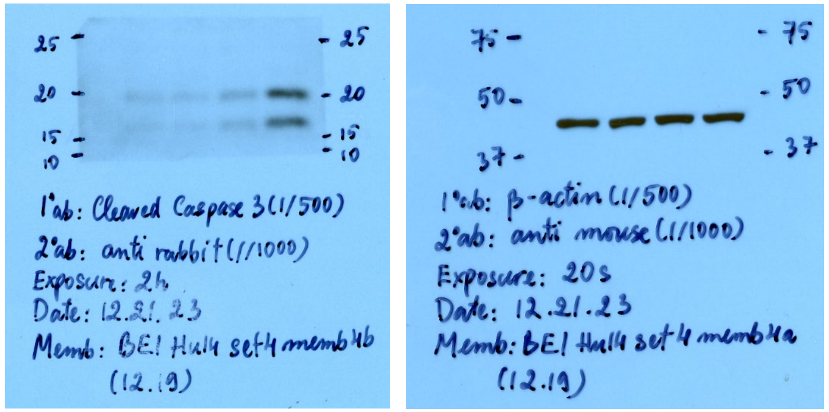

Fig.3F

CHLA15 Cleaved Caspase

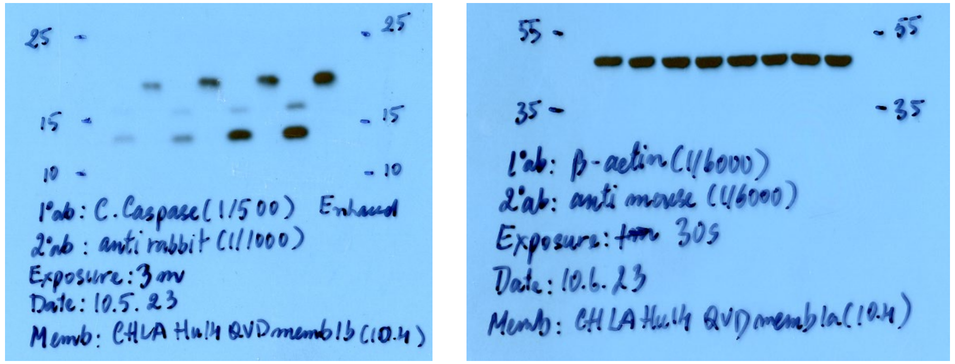

SK-N-BE1 Cleaved Caspase

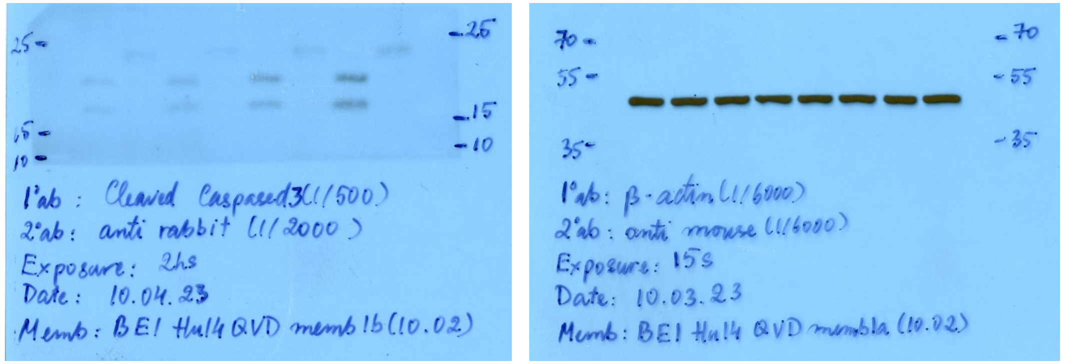

Fig.4A

CHLA15 MLKL

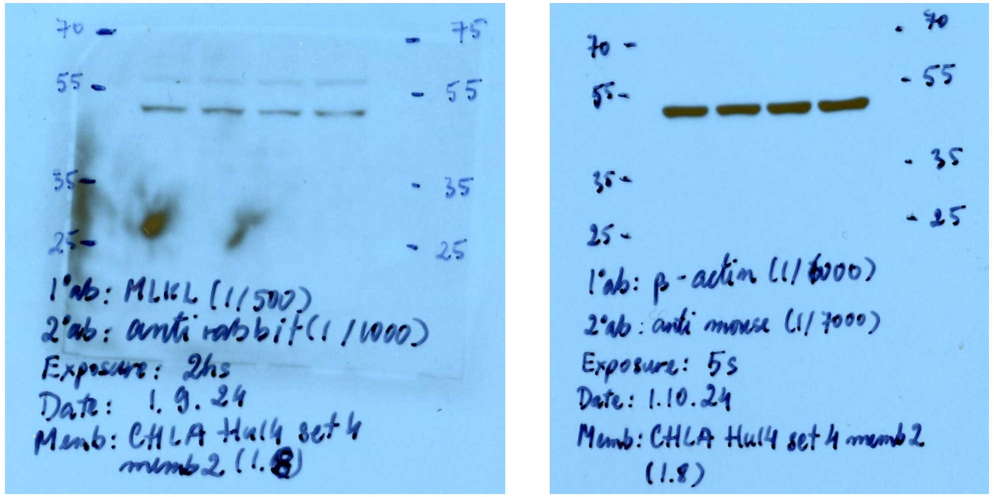

SK-N-BE1 MLKL

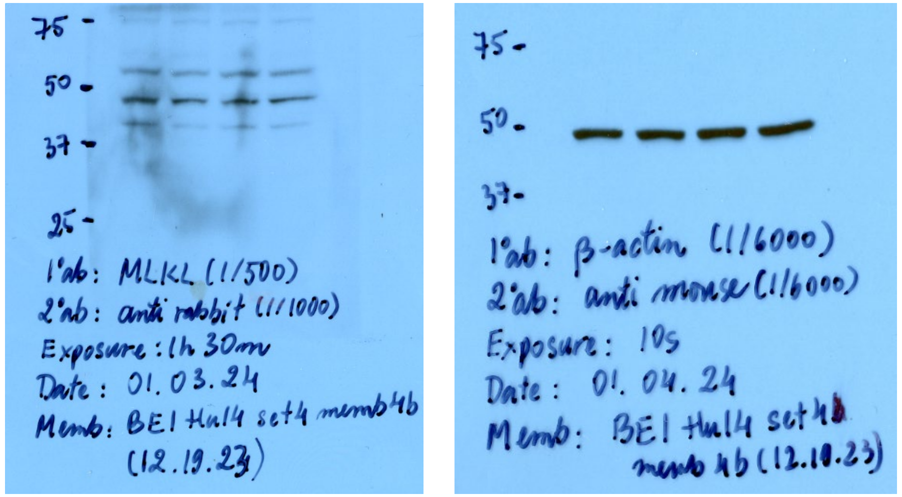

CHLA15 pMLKL

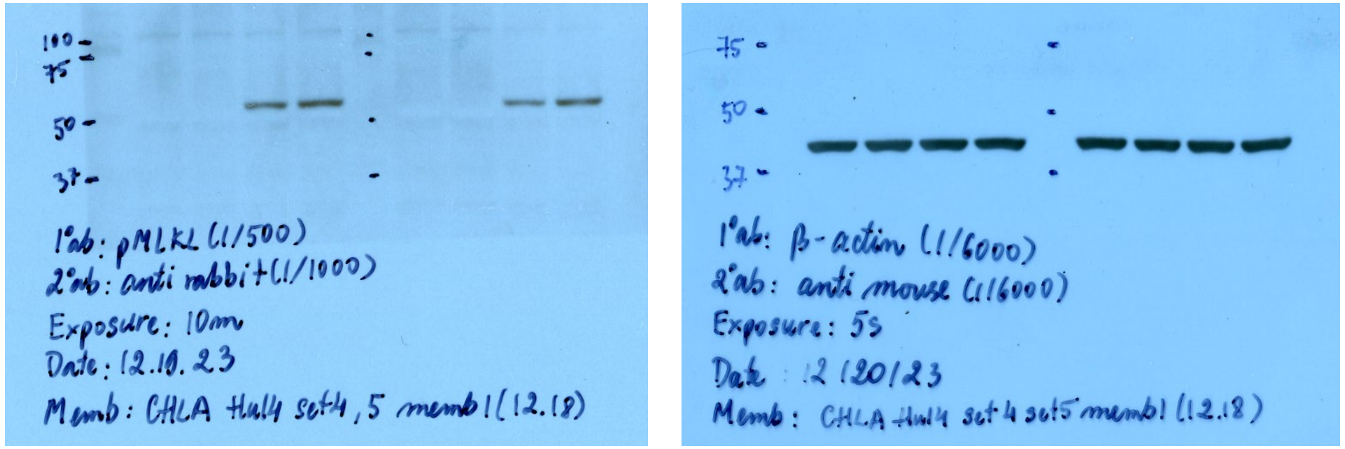

SK-N-BE1 pMLKL

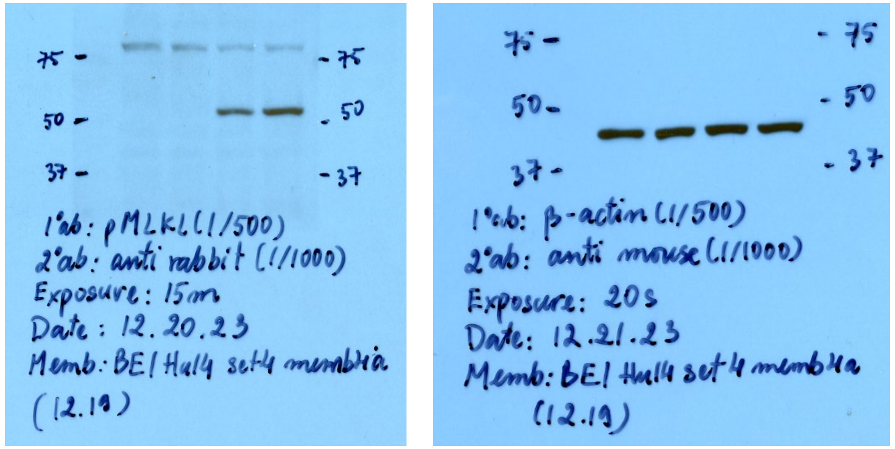

Fig.4B

CHLA15 p62

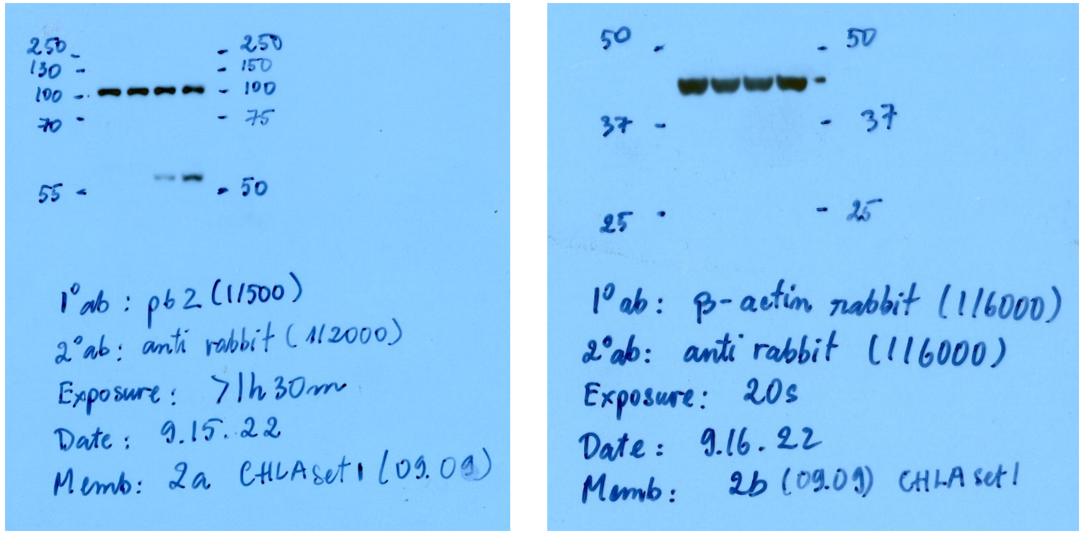

SK-N-BE1 p62

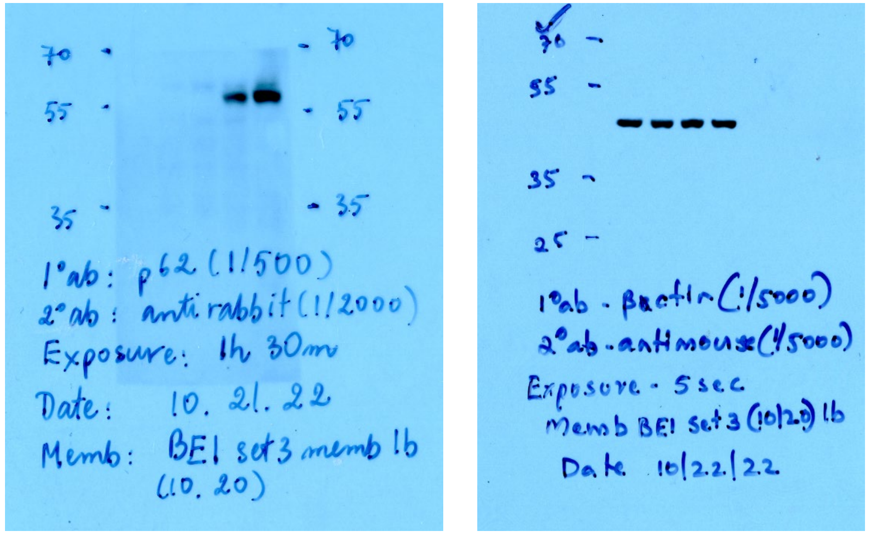

Fig.4C

CHLA15 GPX4

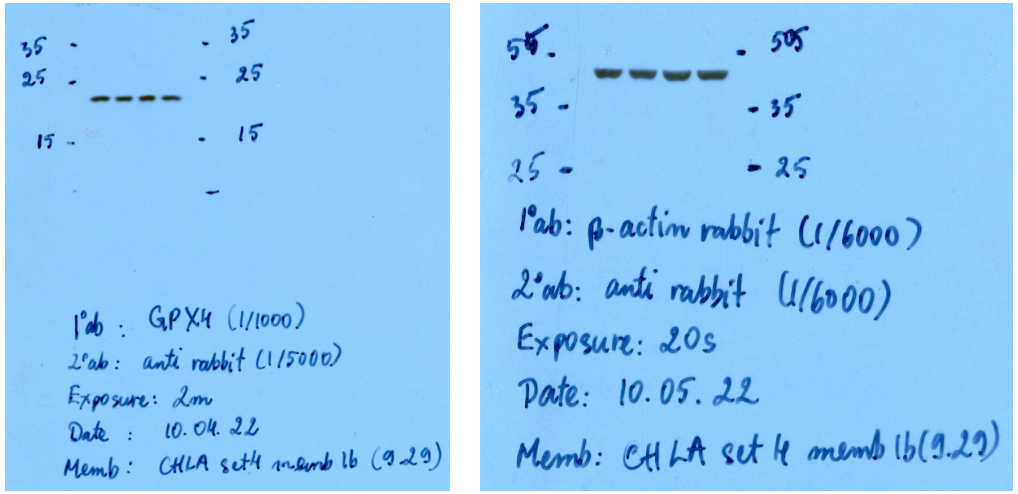

SK-N-BE1 GPX4

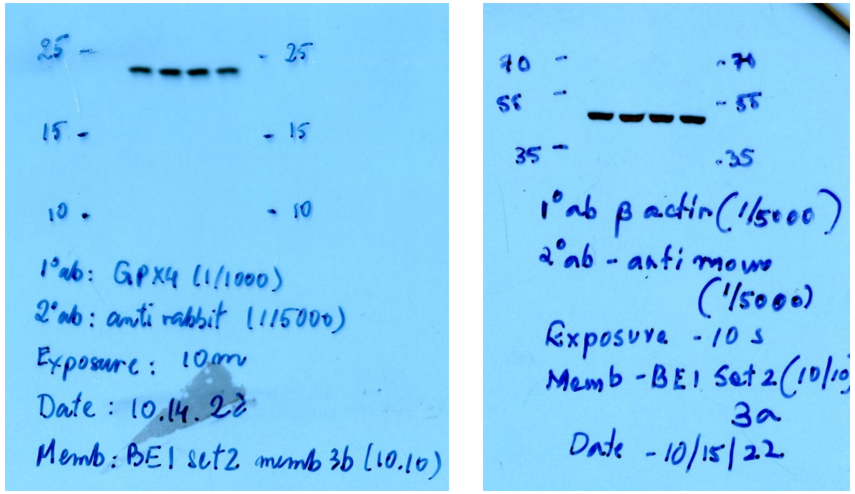

Same membrane used for GSDME and GPX4.
So, same b-actin is presented for Fig 4C and 4D
for SK-N-BE1

Fig.4D

CHLA15 GSDME

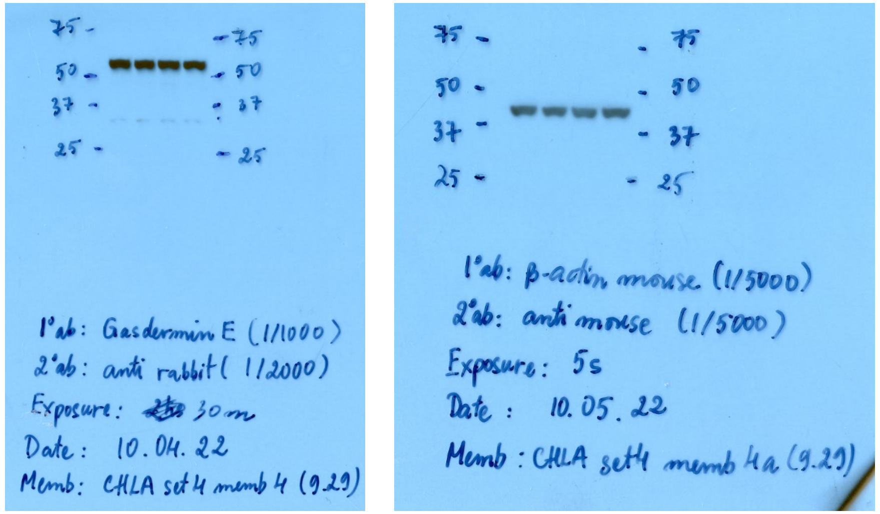

SK-N-BE1 GSDME

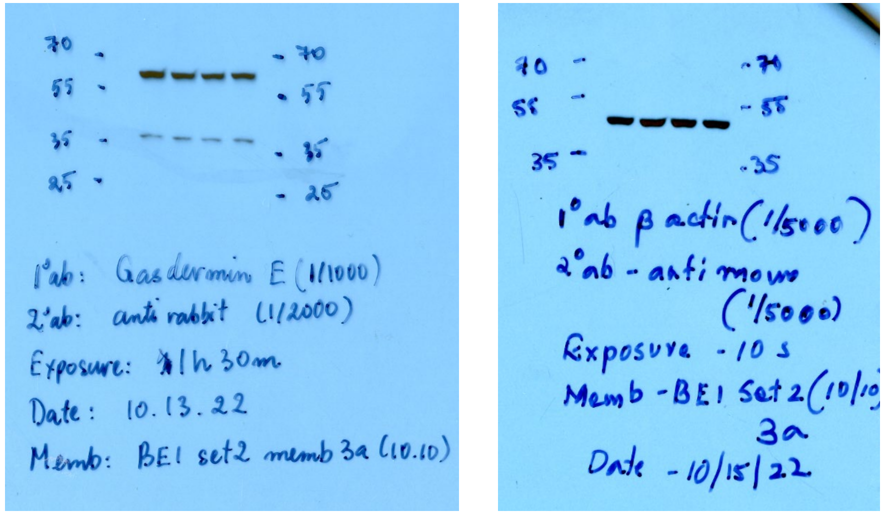

Same membrane used for GSDME and GPX4.
So, same b-actin is presented for Fig 4C and 4D for SK-N-BE1

Fig.S1A

CHLA15 CDKN1

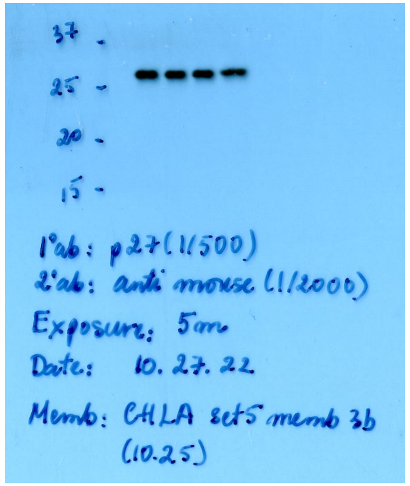

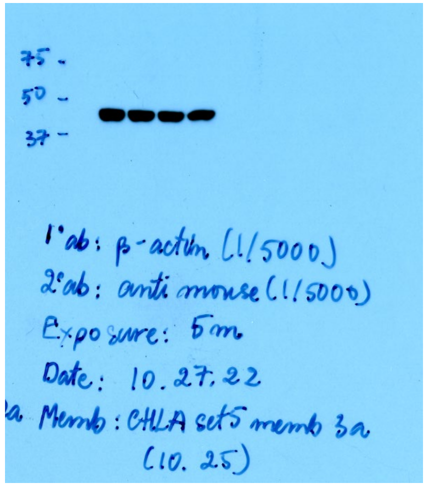

CHLA15 Cyclin D1

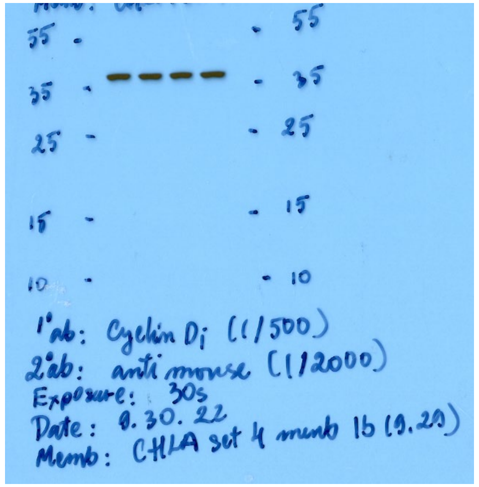

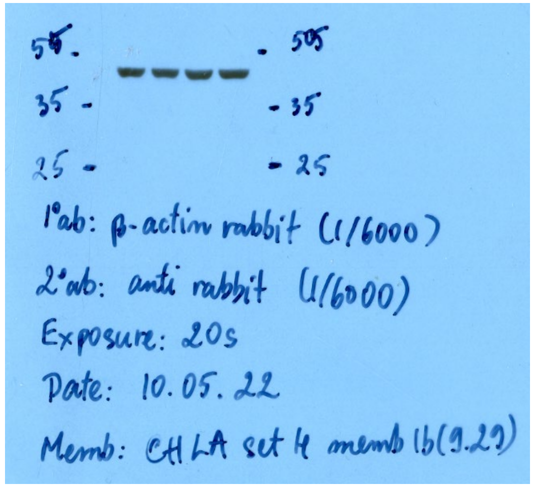

CHLA15 Rb

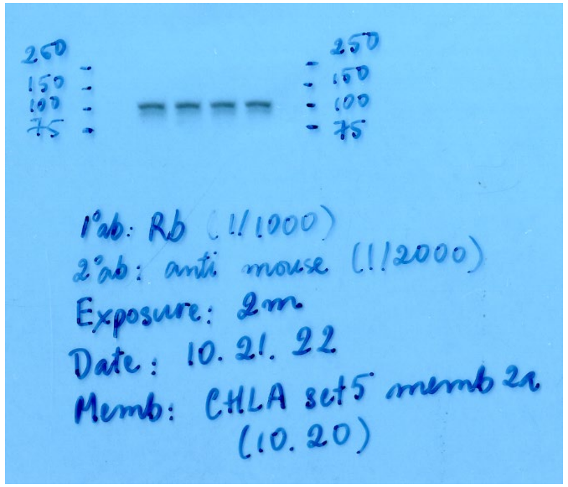

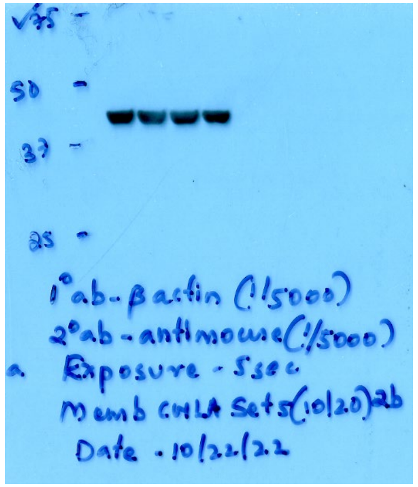

CHLA15 pRb

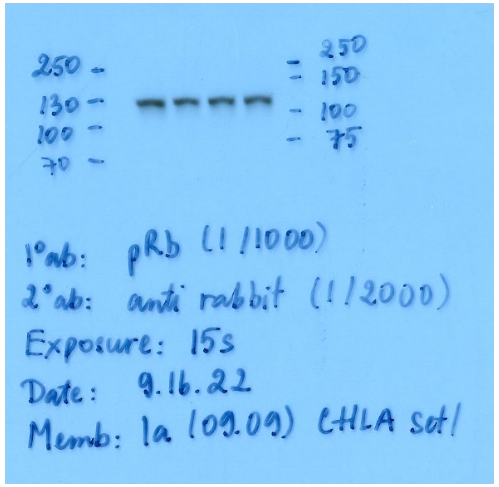

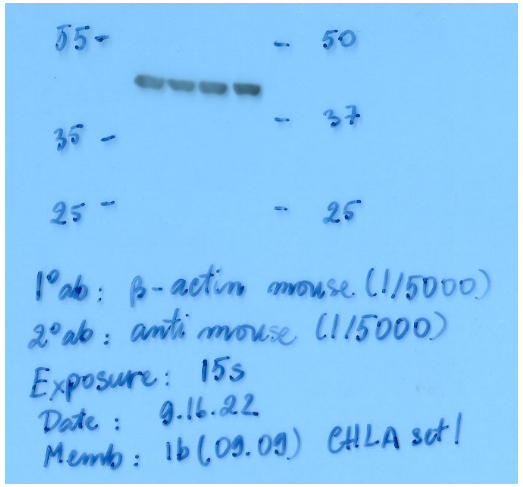

Fig.S1B

SK-N-BE1 CDKN1

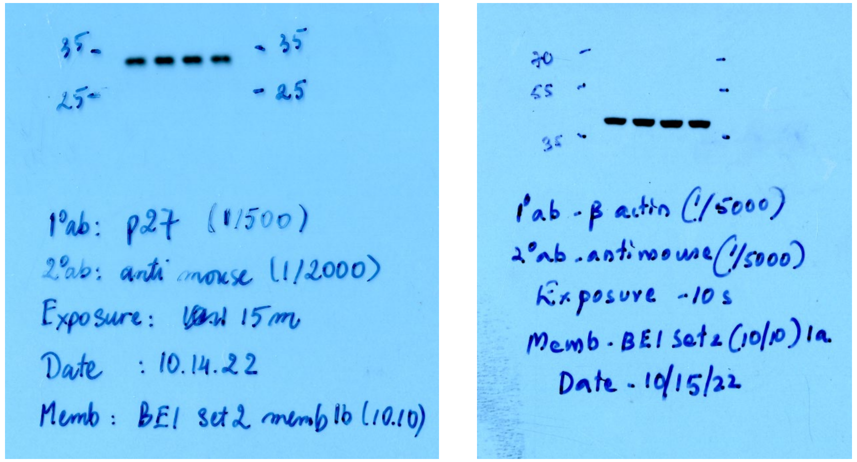

SK-N-BE1 Cyclin D1

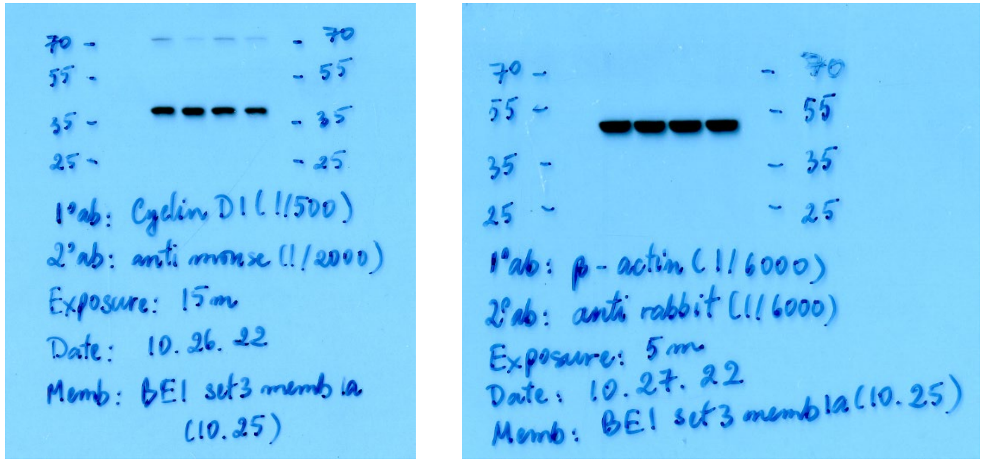

SK-N-BE1 Rb

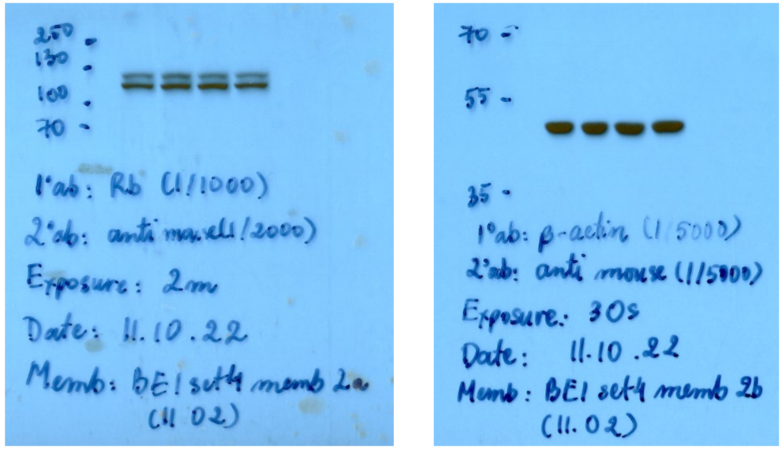

SK-N-BE1 pRb

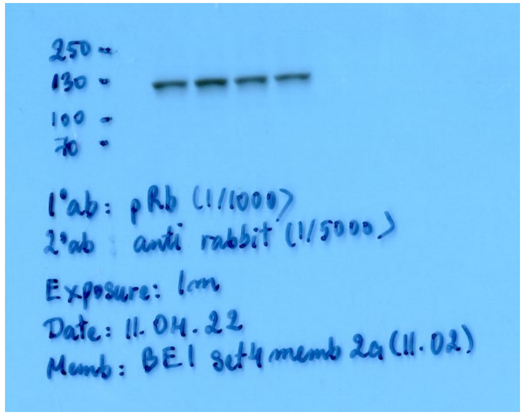

Same b-actin for Rb & pRb

Fig.S2A

WT MLKL

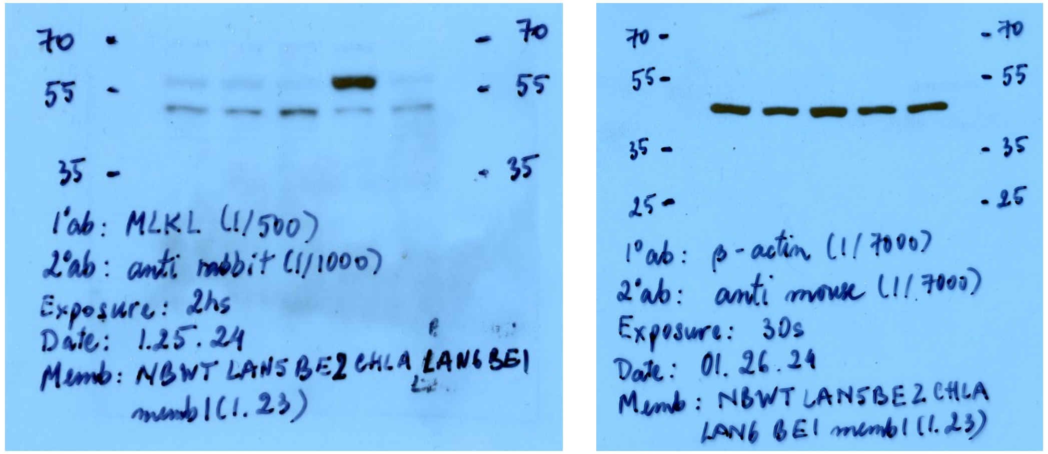

WT RIPK1

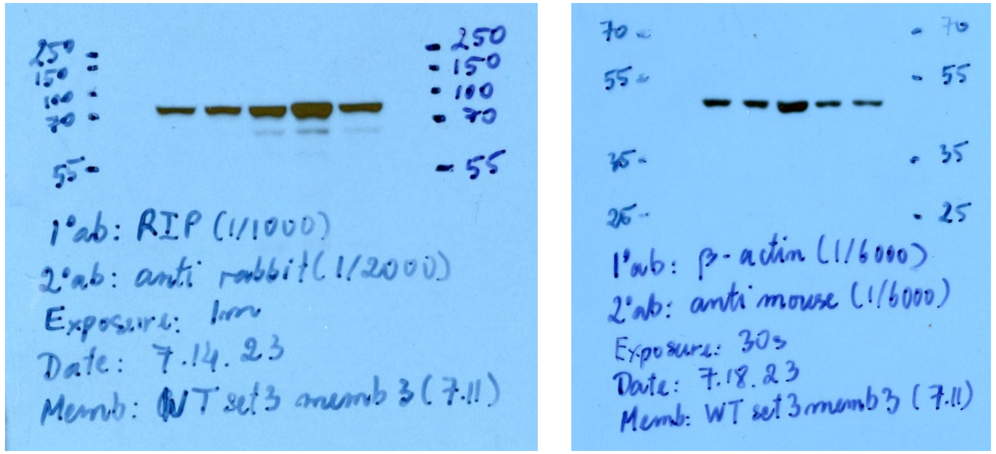

Fig.S2B

CHLA15 RIPK1

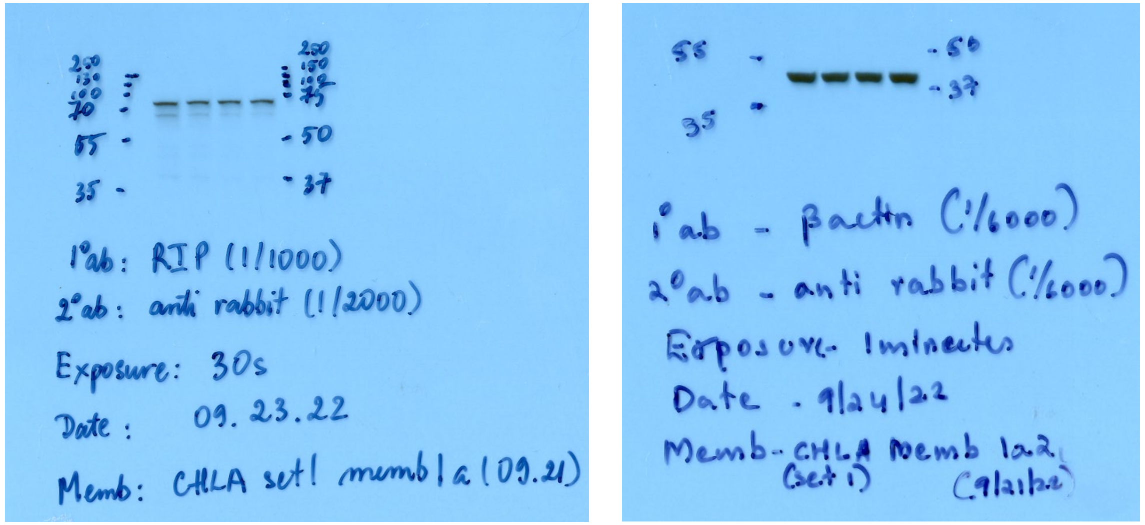

SK-N-BE1 RIPK1

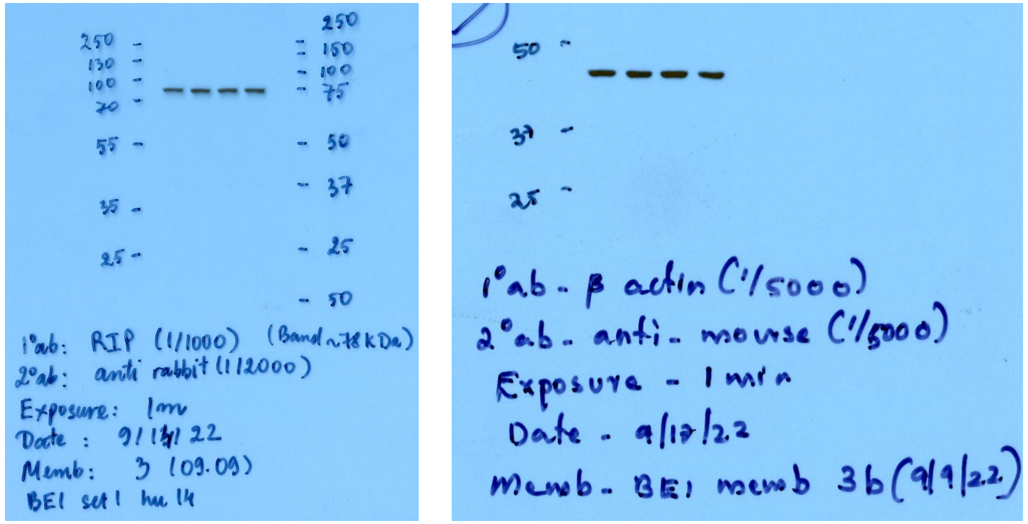

Fig.S2D **CHLA15 LC3B**

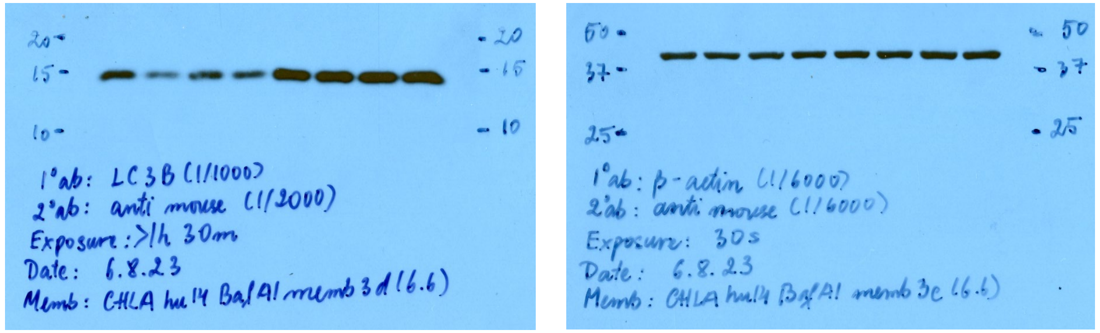

Fig.S2E **SK-N-BE1 LC3B**

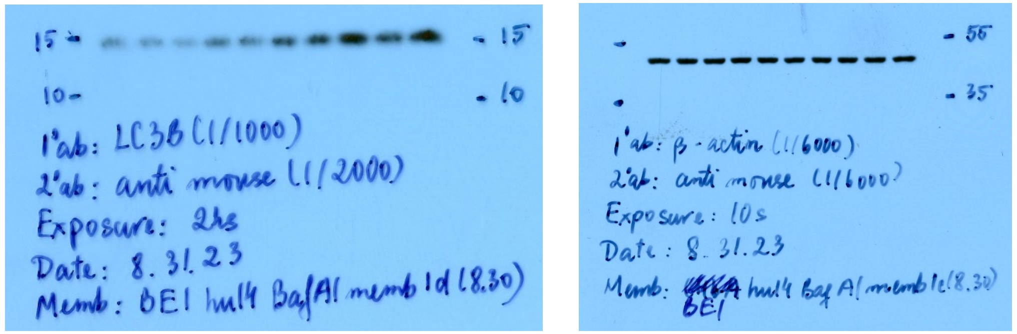

Densitometry intensity ratio

CHLA15

Target protein	Figure #	Replicate	CHLA15	
			IgG	hu14 20μg
p53	2D	1	0.8212	0.8441
		2	0.7166	0.9918
		3	0.7549	0.6152
pp53	2D	1	0.6856	0.4029
		2	0.6095	0.1915
		3	0.6701	0.2570
PARP	3D	1	0.3638	1.2075
		2	0.5962	1.1074
		3	0.6340	0.9031
cleaved PARP	3D	1	0.0441	1.1279
		2	0.0910	0.7330
		3	0.4853	0.8333
cleaved caspase	3D	1	0.2121	1.2348
		2	0.0000	0.9938
		3	0.0831	0.9832
pMLKL	4A	1	0.0000	0.7503
		2	0.0000	0.7399
		3	0.0000	1.0363
p62	4B	1	0.0000	0.9524
		2	0.0000	0.8448
		3	0.0000	0.9902

SK-N-BE1

Target protein	Figure #	Replicate	SK-N-BE1	
			IgG	hu14 20μg
p53	2D	1	0.9226	0.4289
		2	0.8523	0.5944
		3	1.1074	0.6799
pp53	2D	1	0.8093	0.2922
		2	0.7494	0.4113
		3	0.6938	0.4597
PARP	3D	1	1.2114	0.6384
		2	0.9885	0.7571
		3	0.7420	0.9328
cleaved PARP	3D	1	0.0000	0.4147
		2	0.0831	0.8738
		3	0.0699	0.8404
cleaved caspase	3D	1	0.0000	0.3502
		2	0.0000	0.3461
		3	0.0512	0.8615
pMLKL	4A	1	0.1399	0.8183
		2	0.0659	0.7441
		3	0.0608	1.0567
p62	4B	1	0.0000	0.9398
		2	0.0000	0.9381
		3	0.0000	0.9445
