# Supplementary material for: Hu14.18K.322A Causes Direct Cell Cytotoxicity and Synergizes with Induction Chemotherapy in High-Risk Neuroblastoma
Source: Cancers (Basel). 2024 May 30;16(11):2064. doi: 10.3390/cancers16112064 (PMC11171330; doi:10.3390/cancers16112064)
Supplement: Supplementary file 1 [file cancers-16-02064-s001.zip › Supplemental tables revised.pdf]

Supplemental table S1: Primer sequences used in this study.

Name	Sequence (5' to 3')
HPRT-F	TGACACTGGCAAAACAATGCA
HPRT-R	GGTCCTTTTCACCAGCAAGCT
RHOB-F	CTGCTGATCGTGTTCAGTAAGG
RHOB-R	TCAATGTCGGCCACATAGTTC
DACH1-F	ATGTGGAACAAGTTCGCATCC
DACH1-R	TGCAGTCATTGTAGAGGGTCT
PLPP4-F	ATAACATACCTACCCGCCTCAT
PLPP4-R	TCGCCGGATAATTTTCACCAC
DUSP9-F	TTCCGCCAATTTGGAGAGCC
DUSP9-R	TGCTTGTAGTGAAAGTCACCATT
ITGA6-F	CAGTGGAGCCGTGGTTTTG
ITGA6-R	CCACCGCCACATCATAGCC
ITGA5-F	GGCTTCAACTTAGACGCGGAG
ITGA5-R	TGGCTGGTATTAGCCTTGGGT
SDHA-F	TGGGAACAAGAGGGCATCTG
SDHA-R	CCACCACTGCATCAAATTCATG
GSDME-F	ACATGCAGGTCGAGGAGAAGT
GSDME-R	TCAATGACACCGTAGGCAATG
GSDMD-F	GAGTGTGGCCTAGAGCTGG
GSDMD-R	GGCTCAGTCCTGATAGCAGTG
GSDMC-F	TCCGAAATGGTAGGCTACTGT
GSDMC-R	ATGAGGCATTGAAGAGGGTTG
GSDMA-F	GATGTTGGGGACGTACACGAA
GSDMA-R	GGGAGCTTTGCCCTACTCG
GSDMB-F	GCTGAAGAGGGAACTACCCTT
GSDMB-R	TCCTTTACCGTCTCCAGAGTTT
SLC7A11-F	TCTCCAAAGGAGGTTACCTGC
SLC7A11-R	AGACTCCCCTCAGTAAAGTGAC
ST8SIA1 -F	CTATGTGACTCCAAGAGGGAATG
ST8SIA1 -R	CAAGCCAACGCTGAAAGTAAAG
B4GALNT1-F	CAATGGGACACACACCAAATG
B4GALNT1-R	TTACTCACAGGCAAGGGAAAG
ST3GAL5-F	CACAGGTATAGCGTGGACTTAC
ST3GAL5-R	GGTCTGGACTTTACTGGAGAAC
EPAS1-F	GCGCTAGACTCCGAGAACAT
EPAS1-R	TGGCCACTTACTACCTGACCCTT

Supplemental table S2: KEGG pathway enrichment analysis in response to hu14 treatment.

Downregulated pathways in CHLA15

Pathway	Number of genes	Upregulated genes	Downregulated genes	P value
ECM-receptor interaction	62	2	13	9.16E-07

Top 10 upregulated pathways in CHLA15

Pathway	Number of genes	Upregulated genes	Downregulated genes	P value
Oxidative phosphorylation	107	23	1	3.50E-12
Parkinson disease	226	32	5	3.30E-11
Prion disease	213	30	3	1.57E-10
Huntington disease	252	32	5	5.74E-10
DNA replication	35	12	0	1.68E-09
Amyotrophic lateral sclerosis	301	34	9	3.69E-09
Alzheimer disease	308	33	9	2.33E-08
Pathways of neurodegeneration - multiple diseases	385	37	11	5.84E-08
Cell cycle	120	17	2	1.40E-06
Phagosome	86	14	4	2.22E-06

Top 10 upregulated pathways in SK-N-BE1

Pathway	Number of genes	Upregulated genes	Downregulated genes	P value
Phospholipase D signaling pathway	106	21	2	9.11E-09
EGFR tyrosine kinase inhibitor resistance	65	15	4	1.47E-07
MAPK signaling pathway	213	28	6	3.81E-07

Focal adhesion	145	22	5	5.83E-07
ErbB signaling pathway	72	15	3	6.09E-07
Growth hormone synthesis, secretion and action	91	16	3	2.82E-06
Renal cell carcinoma	63	13	4	3.83E-06
mTOR signaling pathway	130	19	6	5.97E-06
cAMP signaling pathway	132	19	3	7.50E-06
Choline metabolism in cancer	78	14	2	9.16E-06

Top 10 downregulated pathways in SK-N-BE1

**Pathway**	**Number of genes**	**Upregulated genes**	**Downregulated genes**	**P value**
Parkinson disease	222	7	91	8.86E-55
Huntington disease	246	15	86	3.73E-45
Prion disease	208	13	78	1.40E-43
Oxidative phosphorylation	106	0	56	1.70E-41
Amyotrophic lateral sclerosis	291	10	88	2.05E-40
Pathways of neurodegeneration - multiple diseases	371	20	96	1.08E-37
Alzheimer disease	303	19	85	2.75E-36
Thermogenesis	190	6	66	1.37E-34
Diabetic cardiomyopathy	156	8	57	2.38E-31
Chemical carcinogenesis - reactive oxygen species	172	9	57	9.07E-29

Supplemental table S3: Genes altered in response to hu14. Top 10 upregulated genes in CHLA15

GENE NAME	Fold change (hu14 vs IgG)
GTF2IP1	7.79
TGFBI	6.63
SOGA3	4.87
PRKCZ-AS1	4.53
TFRC	4.42
LOC124904483	3.21
RNF139-DT	3.17
BTG3-AS1	3.09
TNFSF12-TNFSF13	2.87
LOC107986638	2.82

Top 10 downregulated genes in CHLA15

GENE NAME	Fold change (hu14 vs IgG)
ZNF765-ZNF761	-681.51
LOC105371472	-16.98
LINC02806	-9.74
NIBAN1	-7.30
LOC105376136	-7.22
INHBE	-7.14
LOC124901030	-5.99
FHAD1	-4.52
LOC124900623	-4.51
LOC124904436	-4.28

Top 10 upregulated genes in SK-N-BE1

GENE NAME	Fold change (hu14 vs IgG)
GTF2IP1	6.81
TMED7-TICAM2	6.78
MAF	6.53
LOC124902766	4.53
RNA28SN2	3.99
LOC107987083	3.67
SLX1B-SULT1A4	3.44
SHISA9	3.36
TAF12-DT	3.00
KLKB1	2.92

Top 10 downregulated genes in SK-N-BE1

GENE NAME	Fold change (hu14 vs IgG)
LOC103021295	-279.53
ZNF660-ZNF197	-15.88
BOLA2B	-6.72
GJA9-MYCBP	-6.61
ZNF71-SMIM17	-5.54
ZNF321P	-4.81
LOC124905080	-3.73
LOC100134317	-3.61
BMP8A	-3.22
LOC105371637	-3.21
