# Supplementary figures and images for: Hu14.18K.322A Causes Direct Cell Cytotoxicity and Synergizes with Induction Chemotherapy in High-Risk Neuroblastoma
Source: Cancers (Basel). 2024 May 30;16(11):2064. doi: 10.3390/cancers16112064 (PMC11171330; doi:10.3390/cancers16112064)

**S1A****CHLA15**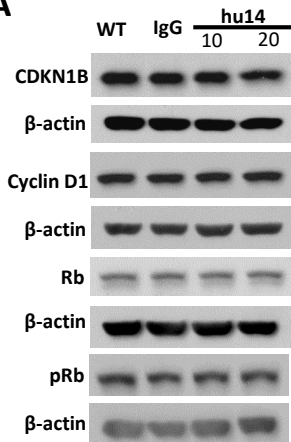**S1B****SK-N-BE1**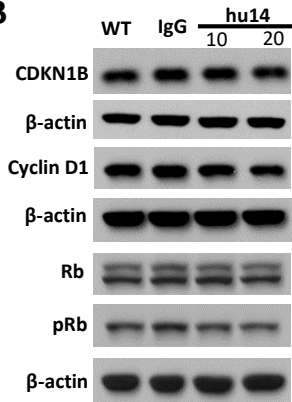

**S2A**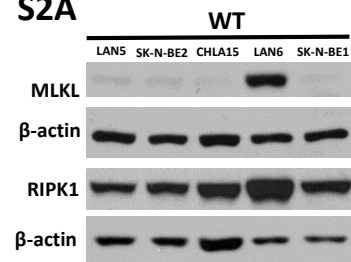**S2B**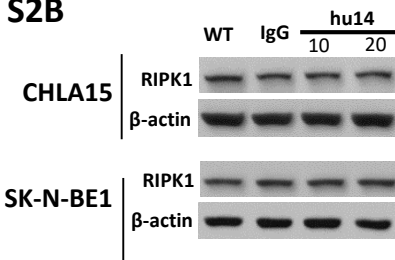**S2D**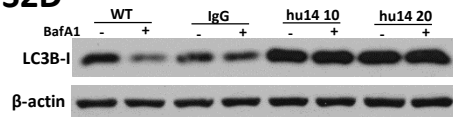**S2E**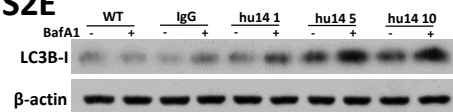**S2C**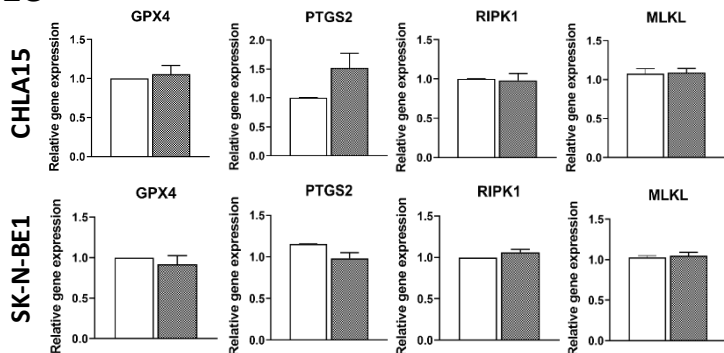**S2F**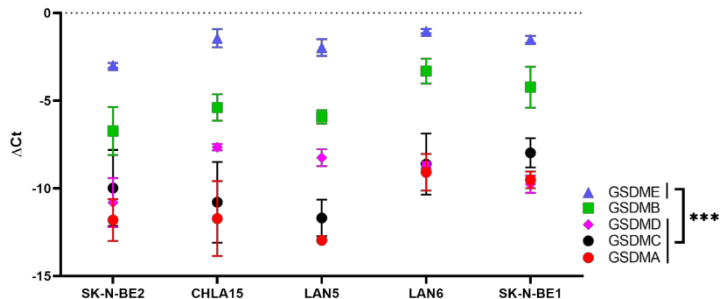

# S3A

## CHLA15

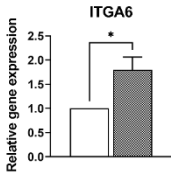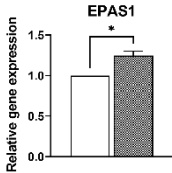

## SK-N-BE1

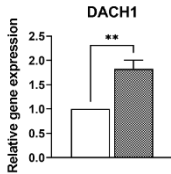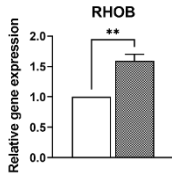

# S3B

## CHLA15

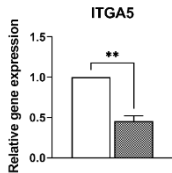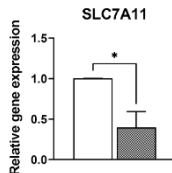

## SK-N-BE1

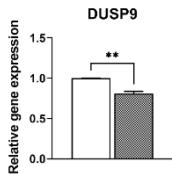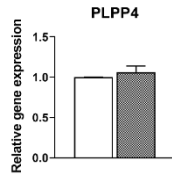

# S4

## B4GALNT1

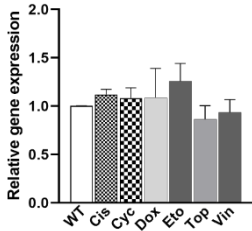

## ST3GAL5

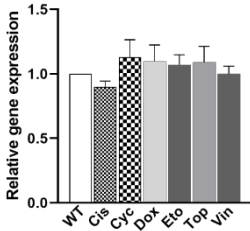

## ST8SIA1

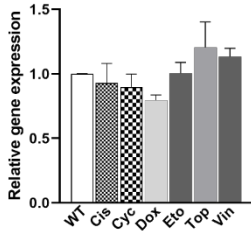

Supplement: Supplementary file 1 [file cancers-16-02064-s001.zip › Combined supplementary Figures.pdf]
